# Genetic Diversity and Population Structure of Basmati Rice (*Oryza sativa* L.) Germplasm Collected from North Western Himalayas Using Trait Linked SSR Markers

**DOI:** 10.1371/journal.pone.0131858

**Published:** 2015-07-28

**Authors:** R. K. Salgotra, B. B. Gupta, Javaid Akhter Bhat, Sandeep Sharma

**Affiliations:** 1 School of Biotechnology, Sher-e-Kashmir University of Agricultural Sciences & Technology of Jammu, Chatha, Jammu (J & K), India; 2 Division of Plant Breeding & Genetics, Sher-e-Kashmir University of Agricultural Sciences & Technology of Jammu, Chatha, Jammu (J & K), India; Wageningen University, NETHERLANDS

## Abstract

One hundred forty one basmati rice genotypes collected from different geographic regions of North Western Himalayas were characterized using 40 traits linked microsatellite markers. Number of alleles detected by the abovementioned primers were 112 with a maximum and minimum frequency of 5 and 2 alleles, respectively. The maximum and minimum polymorphic information content values were found to be 0.63 and 0.17 for the primers RM206 and RM213, respectively. The genetic similarity coefficient for the most number of pairs ranged between of 0.2-0.9 with the average value of 0.60 for all possible combinations, indicating moderate genetic diversity among the chosen genotypes. Phylogenetic cluster analysis of the SSR data based on distance divided all genotypes into four groups (I, II, III and IV), whereas model based clustering method divided these genotypes into five groups (A, B, C, D and E). However, the result from both the analysis are in well agreement with each other for clustering on the basis of place of collection and geographic region, except the local basmati genotypes which clustered into three subpopulations in structure analysis comparison to two clusters in distance based clustering. The diverse genotypes and polymorphic trait linked microsatellites markers in the present study will be used for the identification of quantitative trait loci/genes for different economically important traits to be utilized in molecular breeding programme of rice in the future.

## Introduction

Rice (*Oryza sativa* L.) occupies the premier place among the food crops cultivated around the world; thus rice production and improvement are of interest to the Indian economy. India has the largest acreage under rice (44 million hectares) with annual production of about 104 million tones and ranks second only to China [1]. It provides 43 percent of the caloric requirement for more than 70 per cent of Indian population. Rice protein, though small in amount, is of high nutritional value [2]. Basmati rice makes a metallothionein-like protein, rich in sulfur containing amino acid cysteine that aids in iron absorption. Basmati rice is desirable in international market for its unique quality attributes, such as distinct and pleasant aroma, fluffy texture of cooked rice, high volume expansion during cooking, which is characterized by linear kernel elongation with minimum breadth wise swelling, palatability, easy digestibility and longer shelf life. It is cultivated in the foothills of the Himalayas in the North Western (NW) parts of Indian sub-continent comprising the states of Haryana, Punjab, Uttaranchal, Western Uttar Pradesh, Jammu & Kashmir, Himachal Pradesh and Delhi for hundreds of years. As regards, Jammu & Kashmir it plays an important role in the livelihood of the people of this hilly and sub-mountainous state.

Little attention has been paid to their improvement except for sporadic reports on germplasm evaluation and genetics of some quality traits. As such there is very little information available on genetic diversity of traditional basmati rice. With introduction of high yielding varieties, the land races that include basmati quality types are moving out of cultivation. Moreover, basmati varieties are highly mixed with each other and it is very difficult to differentiate them. Knowledge of genetic diversity and relationships among basmati rice genotypes commonly grown in NW Himalayas may play a significant role in breeding programmes to improve production, productivity, quality traits, biotic and abiotic stresses, and also provide valuable information that can be used by plant breeders as a parental line selection tool. Thus, estimation and quantification of genetic diversity among the basmati rice germplasm are perquisite for their genetic enhancement. Morphological and biochemical markers were used for genetic diversity analysis and for establishing a relationship among cultivars. But these are limited in number, stage specific and highly influenced by the environmental conditions, which thus renders them less popular among the researchers. With the advent of PCR based molecular marker technology, genetic characterization of crop plants has entered into a new era. Amongst various molecular markers, simple sequence repeats (SSR) markers have become a method of choice owing to their high reproducibility, simplicity, easy scoring ability, reliability, co-dominant and multi-allelic nature. Microsatellites or SSR are sequences of a few repeated and adjacent base pairs and abundance throughout the eukaryotic genome [3]. Variations in the number of repeats can be detected by polymerase chain reaction (PCR), with the development of primers (20–30 base pairs) specifically built for amplification and complementary to conserved sequences flanking the microsatellite. These markers have been used for genetic diversity analysis, genotypic identification and population structure estimation in several rice genetic studies [1, 4–24]. It has been hypothesized that the use of random markers for assessing genetic diversity might not reflect the functionally useful variations prevalent at the coding regions of the genome [25], a crucial requisite for the breeding programmes. For suitable selection of suitable diverse parental lines, it is pertinent to study and compare the pattern of genetic diversity by using random vis-à-vis trait-linked simple sequence repeat markers, which would confirm their suitability to assess genetic diversity. Understanding the genetic diversity and structure populations would be vital to association mapping and molecular breeding program in basmati rice.

In the present study, the genetic diversity and population structure of 141 genotypes including landraces, farmer’s varieties, elite cultivars and advanced breeding lines of basmati rice accession collected from NW Himalayas were analyzed using 40 highly polymorphic trait linked SSR markers. Our objectives were to estimate the levels of genetic diversity, and to characterize the population structure of the NW Himalayas basmati germplasm.

## Materials and Methods

### Plant material

**Fig 1:**
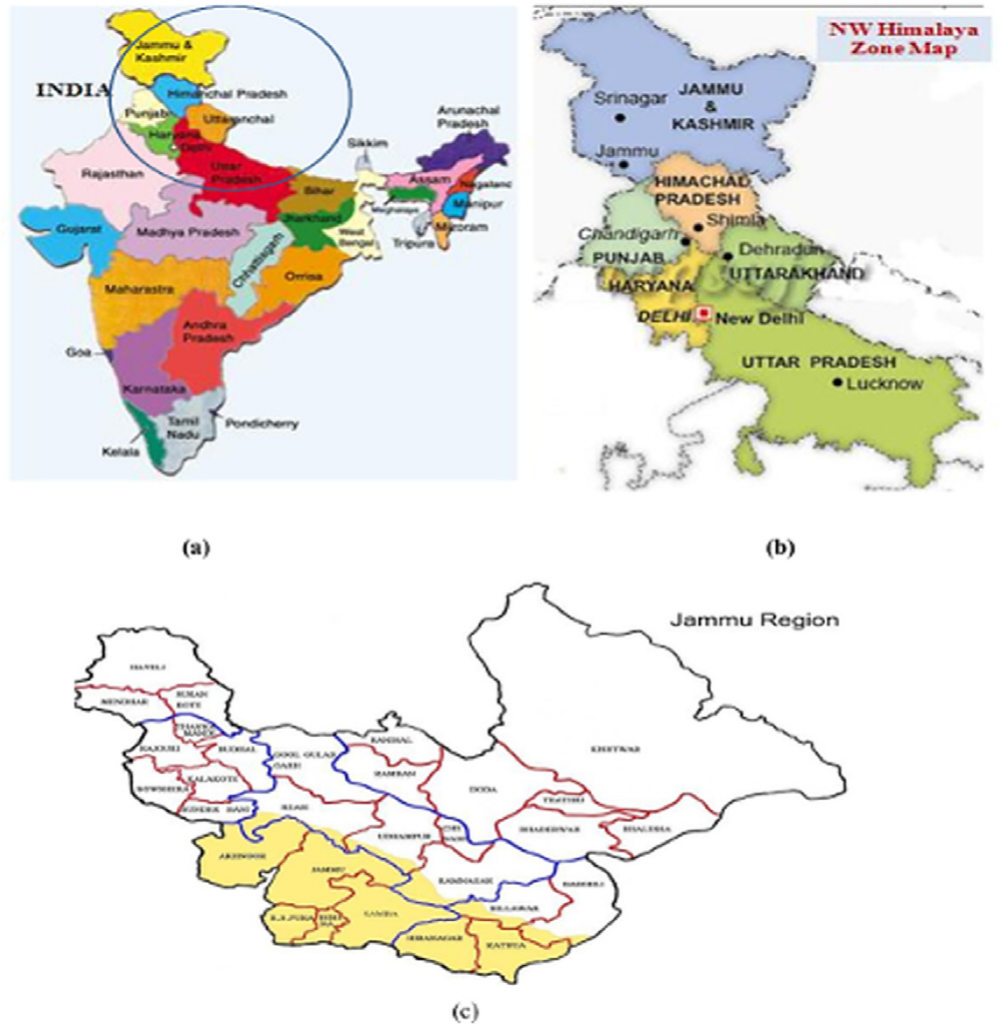
Basmati rice growing areas in NW Himalaya. (a) Political map of India showing the states (encircled) from which basmati rice genotypes were collected. (b) Enlarged view of basmati rice growing states of NW Himalaya. (c) Map of Jammu region showing areas from which local basmati rice (*Oryza sativa* L.) genotypes were collected.

The present study material consisted of 141 basmati rice accessions representing landraces, farmer’s varieties, elite cultivars and advanced breeding lines collected from different basmati growing regions of India (Table 1 and Fig 1). These accessions were planted at Sher-e-Kashmir University of Agricultural Sciences & Technology of Jammu, Chatha, Jammu & Kashmir, India, following panicle to row method to maintain genetic purity. The detailed basic information about the availability of germplasm used in the present study is summarized in S1 and S2 Texts.

**Table 1:** List of basmati rice genotypes used in the study along with their designation, name, place of collection, cluster and subpopulation.

S. No.	Designation	Name of variety/genotype	Place of collection	Cluster	Sub-population
1	SJBR-1	Local Basmati	Nilor, Jammu	I	D
2	SJBR-2	Local Basmati	Arnia, Jammu	I	C
3	SJBR-3	Local Basmati	Badyal, Jammu	II	A
4	SJBR-4	Local Basmati	Badyal, Jammu	II	A
5	SJBR-5	Local Basmati	Badyal, Jammu	II	A
6	SJBR-6	Local Basmati	Barmal, Jammu	II	A
7	SJBR-7	Local Basmati	Biaspur, Jammu	IV	C
8	SJBR-8	Local Basmati	Biaspur, Jammu	III	E
9	SJBR-9	Local Basmati	Bishnah, Jammu	III	E
10	SJBR-10	Local Basmati	Chatha, Jammu	II	A
11	SJBR-11	Local Basmati	Chatha, Jammu	II	A
12	SJBR-12	Local Basmati	Chatha, Jammu	II	A
13	SJBR-13	Local Basmati	Chatha, Jammu	III	E
14	SJBR-14	Local Basmati	Chohalla, R.S.Pura	III	B
15	SJBR-15	Local Basmati	Deoli, Bishnah	II	A
16	SJBR-16	Local Basmati	Bishnah, Jammu	II	A
17	SJBR-17	Local Basmati	Bishnah, Jammu	I	C
18	SJBR-18	Local Basmati	Dhrapti, Jammu	IV	B
19	SJBR-19	Local Basmati	Dhrapti, Jammu	II	A
20	SJBR-20	Local Basmati	Samba	III	E
21	SJBR-21	Local Basmati	Gajola, Kathua	IV	C
22	SJBR-22	Local Basmati	Gajola, Kathua	III	E
23	SJBR-23	Pant Sugandh (Dhan 21)	GBPUAT, Pantnagar, Uttranchal	IV	D
24	SJBR-24	Pant Sugandh (Dhan 15)	GBPUAT, Pantnagar, Uttranchal	IV	D
25	SJBR-25	Pant Sugandh (Dhan 17)	GBPUAT, Pantnagar, Uttranchal	IV	D
26	SJBR-26	Local Basmati	Hansley Chak, Jammu	III	E
27	SJBR-27	Local Basmati	Hansley Chak, Jammu	II	A
28	SJBR-28	Local Basmati	Hansley Chak, Jammu	III	E
29	SJBR-29	Local Basmati	Hansley Chak, Jammu	II	A
30	SJBR-30	Local Basmati	Hansley Chak, Jammu	II	A
31	SJBR-31	Local Basmati	Hansley Chak, Jammu	II	B
32	SJBR-32	Local Basmati	Hansley Chak, Jammu	II	A
33	SJBR-33	Local Basmati	Hansley Chak, Jammu	II	A
34	SJBR-34	Local Basmati	Hansley chak, Jammu	III	E
35	SJBR-35	Pusa Sugandha -3	IARI, New Delhi	I	D
36	SJBR-36	Pusa Sugandha -5	IARI, New Delhi	IV	D
37	SJBR-37	Pusa Sugandha– 2	IARI, New Delhi	IV	D
38	SJBR-38	Pusa 1401	IARI, New Delhi	IV	C
39	SJBR-39	Local Basmati	Sai Kalan Jammu	I	D
40	SJBR-40	Local Basmati	Isharpur, Jammu	IV	C
41	SJBR-41	Local Basmati	Kamoh, Jammu	IV	C
42	SJBR-42	Local Basmati	Kashmir	IV	C
43	SJBR-43	Local Basmati	Kathua	III	E
44	SJBR-44	Local Basmati	Kathua	IV	E
45	SJBR-45	Local Basmati	Kathua	IV	D
46	SJBR-46	Taorori Basmati	Kaul, Haryana	IV	C
47	SJBR-47	Haryana Basmati-1	Kaul, Haryana	IV	C
48	SJBR-48	Haryana Basmati-2	Kaul, Haryana	IV	C
49	SJBR-49	Local Basmati	Ko Brahimna, Samba	III	B
50	SJBR-50	Local Basmati	Ko Brahimna, Samba	II	A
51	SJBR-51	Local Basmati	Ko Brahimna, Samba	II	A
52	SJBR-52	Local Basmati	Ko Brahimna, Samba	II	E
53	SJBR-53	Local Basmati	Ko Brahimna, Samba	IV	B
54	SJBR-54	Local Basmati	Ko Brahimna, Samba	I	D
55	SJBR-55	Local Basmati	Ko Brahimna, Samba	III	E
56	SJBR-56	Local Basmati	Ko Brahimna, Samba	IV	A
57	SJBR-57	Local Basmati	Ko Brahimna, Samba	III	E
58	SJBR-58	Local Basmati	Ko Brahimna, Samba	III	E
59	SJBR-59	Local Basmati	Ko Brahimna, Samba	III	B
60	SJBR-60	Local Basmati	Ko Brahimna, Samba	II	A
61	SJBR-61	Local Basmati	Ko Brahimna, Samba	III	E
62	SJBR-62	Local Basmati	Ko Brahimna, Samba	III	E
63	SJBR-63	Local Basmati	Ko Brahimna, Samba	III	A
64	SJBR-64	Local Basmati	Ko Brahimna, Samba	II	A
65	SJBR-65	Local Basmati	Ko Brahimna, Samba	III	A
66	SJBR-66	Local Basmati	Ko Brahimna, Samba	III	E
67	SJBR-67	Local Basmati	Kogar Basti, Samba	III	B
68	SJBR-68	Local Basmati	Kotha Sainia, Samba	II	A
69	SJBR-69	Local Basmati	Koul, Ramgarh. Samba	III	A
70	SJBR-70	Local Basmati	Koul, Ramgarh. Samba	III	E
71	SJBR-71	Local Basmati	Koul, Ramgarh. Samba	III	E
72	SJBR-72	Local Basmati	Koul, Ramgarh. Samba	III	E
73	SJBR-73	Local Basmati	Koul, Ramgarh. Samba	III	E
74	SJBR-74	Local Basmati	Koul, Ramgarh. Samba	III	E
75	SJBR-75	Local Basmati	Maqala, Bishnah, Jammu	II	B
76	SJBR-76	Local Basmati	Marh, Jammu	III	E
77	SJBR-77	Nagina 22	Meerut, U. P.	I	D
78	SJBR-78	Pakistan Basmati	Pakistan	III	E
79	SJBR-79	Local Basmati	Palampur, H. P.	I	D
80	SJBR-80	PB Basmati– 2	PAU, Punjab	IV	C
81	SJBR-81	PB Basmati– 1	PAU, Punjab	I	D
82	SJBR-82	Basmati 385	PAU, Punjab	I	D
83	SJBR-83	Basmati 386	PAU, Punjab	I	D
84	SJBR-84	PB Local	PAU, Punjab	I	D
85	SJBR-85	PAU 2351	PAU, Punjab	IV	C
86	SJBR-86	PAU 8428	PAU, Punjab	IV	C
87	SJBR-87	PB Mehak	PAU, Punjab	IV	C
88	SJBR-88	Local Basmati	Poonch	IV	C
89	SJBR-89	Local Basmati	Kana Chak, Jammu	IV	B
90	SJBR-90	Local Basmati	Kana Chak, Jammu	III	E
91	SJBR-91	Local Basmati	R. S. Pura, Jammu	II	B
92	SJBR-92	Local Basmati	R. S. Pura, Jammu	III	B
93	SJBR-93	Local Basmati	R. S. Pura, Jammu	III	E
94	SJBR-94	Local Basmati	R. S. Pura, Jammu	II	A
95	SJBR-95	Local Basmati	R. S. Pura, Jammu	III	A
96	SJBR-96	Local Basmati	R. S. Pura, Jammu	IV	A
97	SJBR-97	Local Basmati	R. S. Pura, Jammu	IV	C
98	SJBR-98	Local Basmati	R. S. Pura, Jammu	II	B
99	SJBR-99	Local Basmati	R. S. Pura, Jammu	IV	C
100	SJBR-100	Local Basmati	R. S. Pura, Jammu	III	E
101	SJBR-101	Local Basmati	R. S. Pura, Jammu	III	A
102	SJBR-102	Local Basmati	R. S. Pura, Jammu	II	A
103	SJBR-103	Local Basmati	R. S. Pura, Jammu	III	A
104	SJBR-104	Local Basmati	R. S. Pura, Jammu	III	A
105	SJBR-105	Local Basmati	R. S. Pura, Jammu	II	A
106	SJBR-106	Local Basmati	R. S. Pura, Jammu	IV	A
107	SJBR-107	Local Basmati	R. S. Pura, Jammu	III	E
108	SJBR-108	Local Basmati	R. S. Pura, Jammu	III	E
109	SJBR-109	Local Basmati	R. S. Pura, Jammu	I	D
110	SJBR-110	Local Basmati	R. S. Pura, Jammu	IV	C
111	SJBR-111	Local Basmati	R. S. Pura, Jammu	IV	C
112	SJBR-112	Local Basmati	R. S. Pura, Jammu	III	E
113	SJBR-113	Local Basmati	Rajpura Samba	I	C
114	SJBR-114	Local Basmati	Ramgarh. Jammu	III	E
115	SJBR-115	Local Basmati	Ramgarh. Jammu	III	A
116	SJBR-116	Local Basmati	Ramgarh. Jammu	II	A
117	SJBR-117	Local Basmati	Ramgarh. Jammu	III	B
118	SJBR-118	Local Basmati	Ramgarh. Jammu	III	B
119	SJBR-119	Local Basmati	Ramgarh. Jammu	III	B
120	SJBR-120	Local Basmati	Ramgarh. Jammu	III	B
121	SJBR-121	Local Basmati	Rohi Morh, Jammu	IV	B
122	SJBR-122	Local Basmati	Sainia, Jammu	III	E
123	SJBR-123	Local Basmati	Sainia, Jammu	III	E
124	SJBR-124	Local Basmati	Sajadpura Jammu	III	E
125	SJBR-125	Local Basmati	Salma Chak, Jammu	III	B
126	SJBR-126	Local Basmati	Samba Jammu	III	E
127	SJBR-127	Local Basmati	Sanora, Samba	IV	A
128	SJBR-128	Local Basmati	Sarore, Samba	III	B
129	SJBR-129	Local Basmati	Sarore, Samba	II	A
130	SJBR-130	Local Basmati	Shahpur, Jammu	III	E
131	SJBR-131	Local Basmati	Shahpura Jammu	III	E
132	SJBR-132	SJR– 81	SKUAST-J	IV	D
133	SJBR-133	Pusa 1121	SKUAST-J	IV	C
134	SJBR-134	Basmati -370	SKUAST-J	IV	C
135	SJBR-135	RR– 600	SKUAST-J	IV	C
136	SJBR-136	Basmati-564	SKUAST-J	IV	C
137	SJBR-137	Ranbir Basmati	SKUAST-J	IV	C
138	SJBR-138	Sanwaal Basmati	SKUAST-J	III	E
139	SJBR-139	Basmati 370	SKUAST-J	III	E
140	SJBR-140	SJR 242	SKUAST-J	IV	C
141	SJBR-141	Local Basmati	Tasava, Jammu	III	E

### DNA extraction

Two grams fresh leaf samples were collected from each genotype for DNA extraction. Total genomic DNA was isolated from each genotype by CTAB method [26]. Quantification of DNA samples was done by using the Nanodrop (mySPEC, Scientific GmbH, Germany). The quality of the DNA was estimated by using 0.8% agarose gel electrophoresis. High concentration of DNA samples was further diluted in 10:1 Tris-EDTA to a working concentration of 50 ng/μl and stored at 4°C for PCR based marker analysis.

### PCR assay

PCR amplification was performed on each of the 141 basmati rice genotypes using primers for each SSR locus. Total 40 pairs of rice primers flanking the microsatellite region were selected from previously developed and published [27]. Detailed description of the primers is available at www.gramene.org/markers/microsat/. The primer pair was selected from each chromosome. PCR reaction was prepared with 50 ng of rice genomic DNA, 0.2 μg of 3’ and 5’ end primers, 200 mM of each dNTP, 10X PCR buffer containing 50 mM KCL, 10 mM Tris HCl (pH 8.9), 2.0 mM MgCl_2_ and one unit of *Taq* polymerase with a total of 25 μL solutions individually for all 40 primer pairs. PCR thermal cycler was programmed as one step at 94°C for 4min, followed by 1 min at 94°C, 1 min and 30 seconds at 55°C, 1 min at 72°C and a final cycle of 10 min at 72°C. PCR amplification with each primer was performed thrice and only reproducible and distinct bands were scored and subjected to analysis. Amplified products were separated on 3.5% of agarose gel followed by staining with ethidium bromide. A 100-bp DNA ladder (Life Technologies-GIBCO BRL) was used to estimate the size of each band.

### SSR marker analysis

Amplified fragments of different sizes were considered as different alleles. DNA bands that were amplified by a given primer were scored as present (1) or absent (0) for all the samples under study. In order to determine the utility of these markers, number of amplicons/alleles per marker, major allele frequency, polymorphic information content (PIC), effective multiplex ratio (EMR) / resolving power (RP), discrimination power (DP) and marker index (MI) were calculated. The polymorphic information content values of individual primer were calculated based on the formula PIC = 1- Σ^n^_i = 1_ P^2^_ij_ [27]. Marker index, a product of information content, as measured by PIC and EMR was calculated [3]. Resolving power (RP) and discrimination power (DP) of each primer combination were calculated using standard methods [28, 29]. The Jaccard’s similarity index was calculated using NTSYS-pc version 2.02e (Applied Bio-Statistics, Inc., Setauket, NY, USA) package to compute pairwise Jaccard’s similarity coefficients [30] and this similarity matrix was used in cluster analysis using an unweighted pair-group method with arithmetic averages (UPGMA) and sequential, agglomerative, hierarchical and nested (SAHN) clustering algorithm to obtain a dendrogram. The genetic similarity coefficient was calculated for each pair of genotypes [31] to determine the effectiveness of the SSR loci in distinguishing each of the 141 genotypes.

### Population structure analysis

Model based cluster analysis was performed to infer genetic structure and to define the number of clusters (gene pools) in the dataset using the software STRUCTURE version 2.3.4 [32]. The number of presumed populations (K) was set from 2 to 10, and the analysis was repeated 5 times. We used the burn-in period of 50,000 and Monte Carlo Markov Chain replicates of 100,000 and a model without admixture and correlated allele frequencies was used [33]. The run with maximum likelihood was used to assign individual genotypes into groups. Within a group, genotypes with affiliation probabilities (inferred ancestry) ≥80% were assigned to a distinct group and those with <80% were treated as “admixture”, i.e., these genotypes seem to have a mixed ancestry from parents belonging to different gene pools or geographical origins. The significance of population differentiation clustered by STRUCTURE 2.3.4 was further investigated by performing an analysis of molecular variance (AMOVA) with Arlequin 3.5 [34]. Pairwise population differentiation was estimated among five sub-populations using Arlequin 3.5 [34]. Another dendrogram among the five subpopulation (generated through Structure analysis) based on unbiased genetic distance [35] was constructed by UPGMA (unweighted pair-group method with arithmetic average) using POPGENE version 1.31.

## Results

### SSR Polymorphism among basmati rice varieties

**Fig 2:**
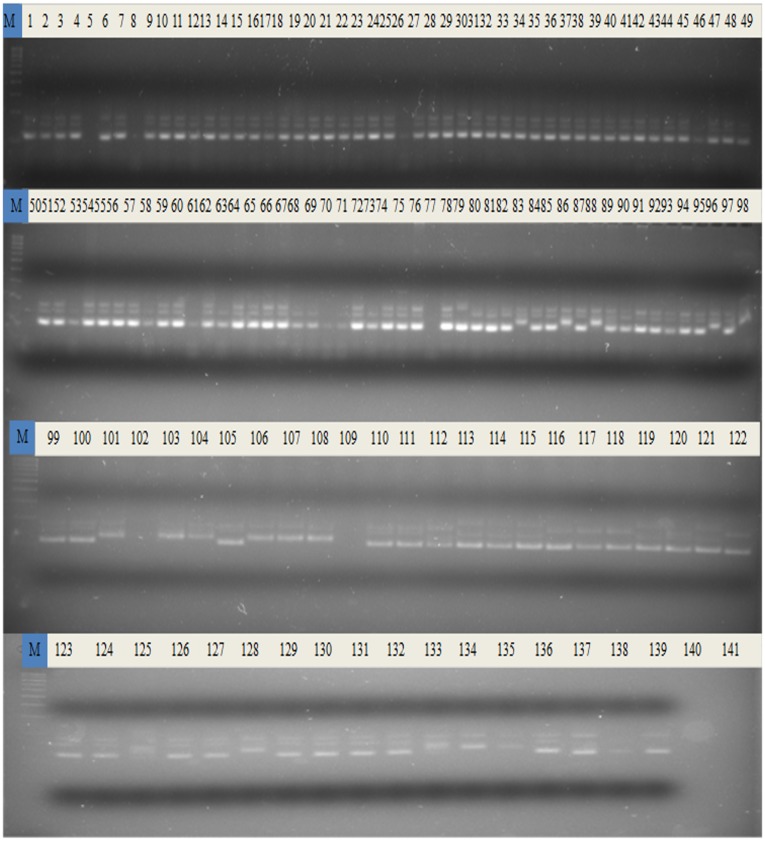
Gel picture of marker RM212 showing banding pattern in 141 basmati rice genotypes.

**Fig 3:**
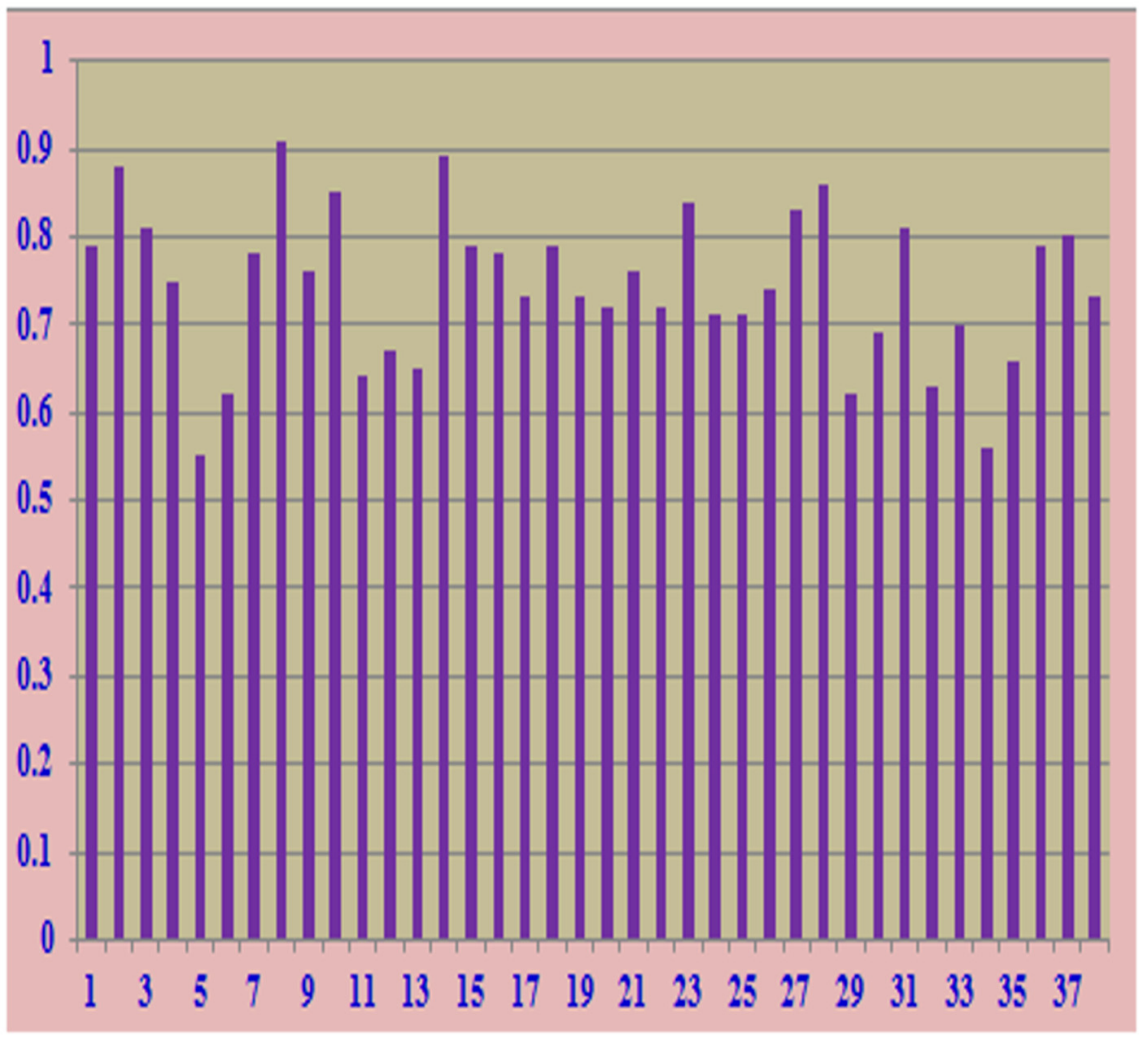
Major allele frequency of polymorphic SSR markers.

All the 141 basmati rice accessions were genotyped with 40 traits linked microsatellite markers; and were selected for their ability to produce amplified product at optimum concentration, polymorphism level among the varieties and consistency of the pattern. Out of 40 traits linked microsatellite markers, two markers (RM130 and RM571) were found monomorphic revealing one allele at each locus in all the genotypes. Total 114 alleles were scored from these primer pairs, and 95 percent were found polymorphic. The gel picture showing banding pattern of 141 genotypes of basmati rice with RM3 marker is given in Fig 2. These loci were used to discriminate the morphologically similar genotypes; their use allowed to discriminate all the genotypes. The respective values for overall genetic variability for polymorphism information content, resolving power, major allele frequency, discrimination power and marker index across all the 141 genotypes are given in Table 2. Highest PIC value (0.63) was observed for the primer RM206 and lowest PIC value (0.17) was recorded for the primer RM213 (Table 2) with an average of 0.405. The MI values ranged from 3.14 to 0.34 with an average of 1.22. The RP is a feature of marker that indicates the discriminatory potential of the primer. RP ranged from 1.76 to 0.34 with an average of 1.01 for polymorphic marker. In case of polymorphic markers the major allele frequency ranged from 0.55 to 0.91 with an average of 0.74 (Table 2 and Fig 3). The DP values ranged from 0.62 to 0.16 with an average of 0.41. The allele number per locus varied from 2 to 5 with an average of 3 alleles per locus (Table 2).

**Table 2:** List of markers used, chromosome number, functional gene, associated trait, number of alleles, major allele frequency, PIC values, marker index (MI), resolving power (RP), and discrimination power (DP).

S. No.	Marker	Chromosome no.	Functional gene	Associated trait	No. of Alleles	Major Allele frequency	PIC value	Marker index	Resolving power	Discrimination power
1	RM1	1	*qGW-1*	grain width	4	0.79	0.36	1.45	0.82	0.36
2	RM5	1	*yld1*.*1*	yield per plot	2	0.88	0.22	0.43	0.46	0.21
3	RM212	1	*gw1*.*1*	grain weight	3	0.81	0.32	0.91	0.74	0.32
4	RM302	1	*gw1*.*1*	grain weight	5	0.75	0.42	2.09	0.96	0.42
5	RM472	1	*gpl1*.*1*	grains per plant	3	0.55	0.55	1.65	1.76	0.54
6	RM145	2	*GW2*	grain width	5	0.62	0.53	2.62	1.48	0.53
7	RM208	2	*gw2*.*1*	grain weight	2	0.78	0.35	0.7	0.86	0.34
8	RM213	2	*gpl2*.*1*	grains per plant	2	0.91	0.17	0.34	0.34	0.16
9	RM262	2	*np2*.*1*	Panicles per plant	3	0.76	0.61	1.81	0.94	0.39
10	RM263	2	*Ftg-1*	tillering	3	0.85	0.27	0.81	0.56	0.25
11	RM2634	2	*GW2*	grain width	3	0.64	0.53	0.53	1.42	0.53
12	RM5897	2	*GW2*	grain width	2	0.67	0.45	0.9	1.3	0.44
13	RM6318	2	*GW2*	grain width	4	0.65	0.52	2.09	1.36	0.52
14	RM411	3	*Gs3*	grain size	2	0.89	0.2	0.37	0.42	0.18
15	RM520	3	*gw3*.*1*	grain weight	5	0.79	0.37	1.83	0.8	0.34
16	RM3646	3	*Gs3*	grain size	3	0.78	0.36	1.07	0.84	0.35
17	RM252	4	*qpn4*.*4*	Panicles per hill	3	0.73	0.43	1.29	1.06	0.43
18	RM273	4	*qpn4*.*4*	Panicles per hill	2	0.79	0.34	0.7	0.82	0.32
19	RM303	4	*pss4*.*1*	percent seed set	2	0.73	0.4	0.8	1.06	0.39
20	RM16	5	*qSW5*	seed width	2	0.72	0.41	0.82	1.1	0.4
21	RM17	5	*qSW5*	seed width	3	0.76	0.4	1.19	0.94	0.39
22	RM26	5	*qGW-5*	grain width	2	0.72	0.41	0.82	1.1	0.4
23	RM289	5	*qGW5*	grain width	3	0.84	0.28	0.83	0.6	0.27
24	RM3	6	*Moc1*	tillering	3	0.71	0.46	1.38	1.12	0.45
25	RM70	7	*Ghd7*	grains per panicle, plant height, heading date	4	0.71	0.47	1.89	1.1	0.46
26	RM5436	7	*Ghd7*	grains per panicle, plant height, heading date	2	0.74	0.4	0.78	1.02	0.38
27	RM5499	7	*Ghd7*	grains per panicle, plant height, heading date	2	0.83	0.29	0.57	0.66	0.27
28	RM201	9	*gw9*	grain weight	2	0.86	0.34	0.49	0.54	0.24
29	RM205	9	*gw9*.*2*	grain weight	3	0.62	0.54	1.63	1.48	0.54
30	RM228	10	*gw10b*	grain weight	3	0.69	0.45	1.35	1.22	0.45
31	RM4	11	*gw11*	grain weight	2	0.81	0.32	0.63	0.74	0.31
32	RM20	11	*gw11*.*1*	grain weight	2	0.63	0.47	0.95	1.35	0.47
33	RM202	11	*ppl11*.*1*	Panicles per plant	4	0.7	0.48	2.41	1.16	0.47
34	RM206	11	*qGW-11-1*	grain size	5	0.56	0.63	3.14	1.7	0.62
35	RM209	11	*gw11*	grain weight	4	0.66	0.53	2.09	1.34	0.52
36	RM nksrssr04-11	4	*gw*	grain weight	3	0.79	0.36	1.07	0.82	0.34
37	RM190	6	*AC*,*GC*,*GT*	Amylose content, gel consistency, gelatinisation temperature	3	0.8	0.35	1.03	0.74	0.32

Functional genes as modified from [25], [36], [37], [38], [39].

### Genetic relationship

**Fig 4:**
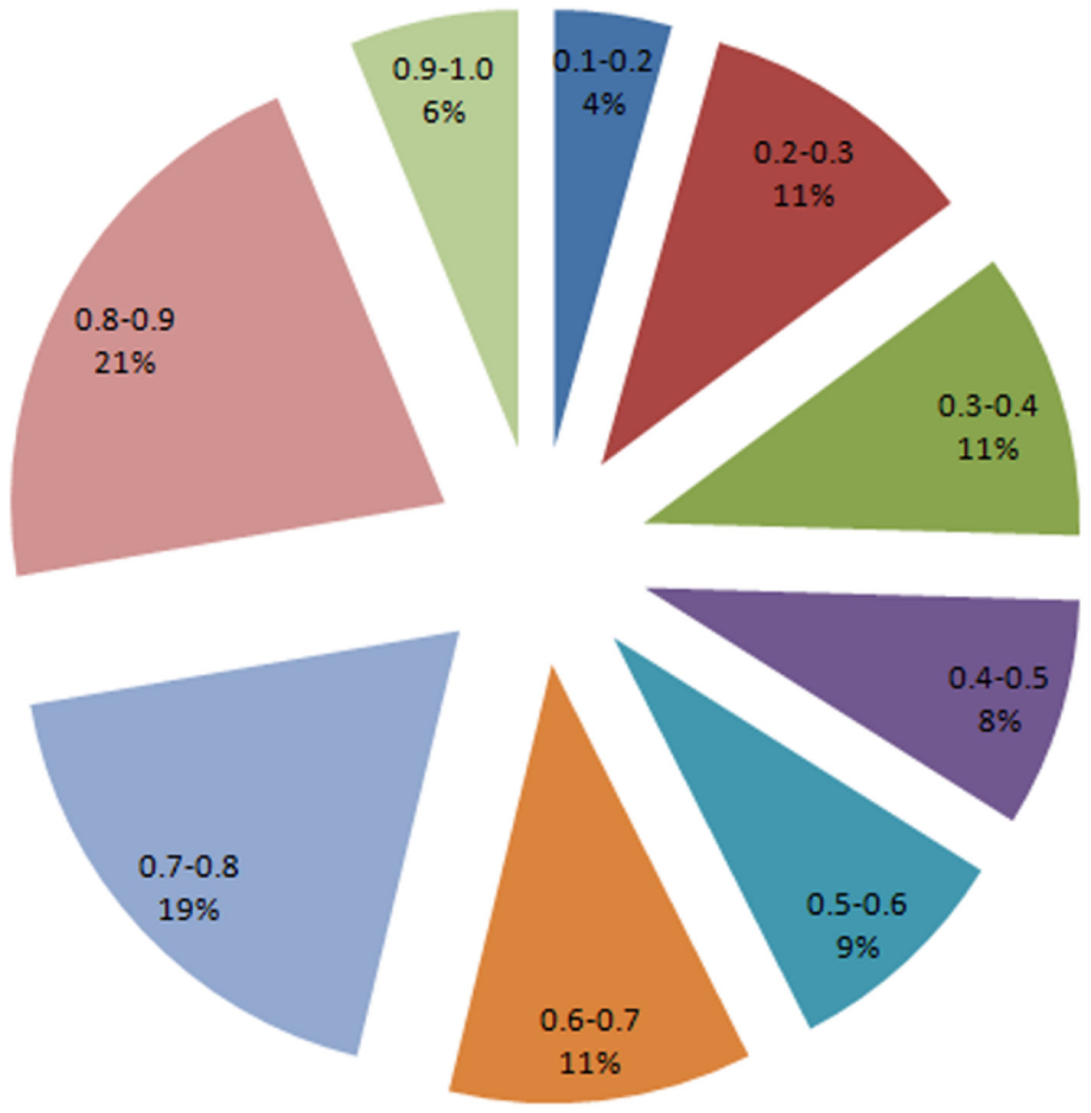
Percentage of distribution of the genetic similarity coefficient calculated between a 9870 pair of genotypes.

**Fig 5:**
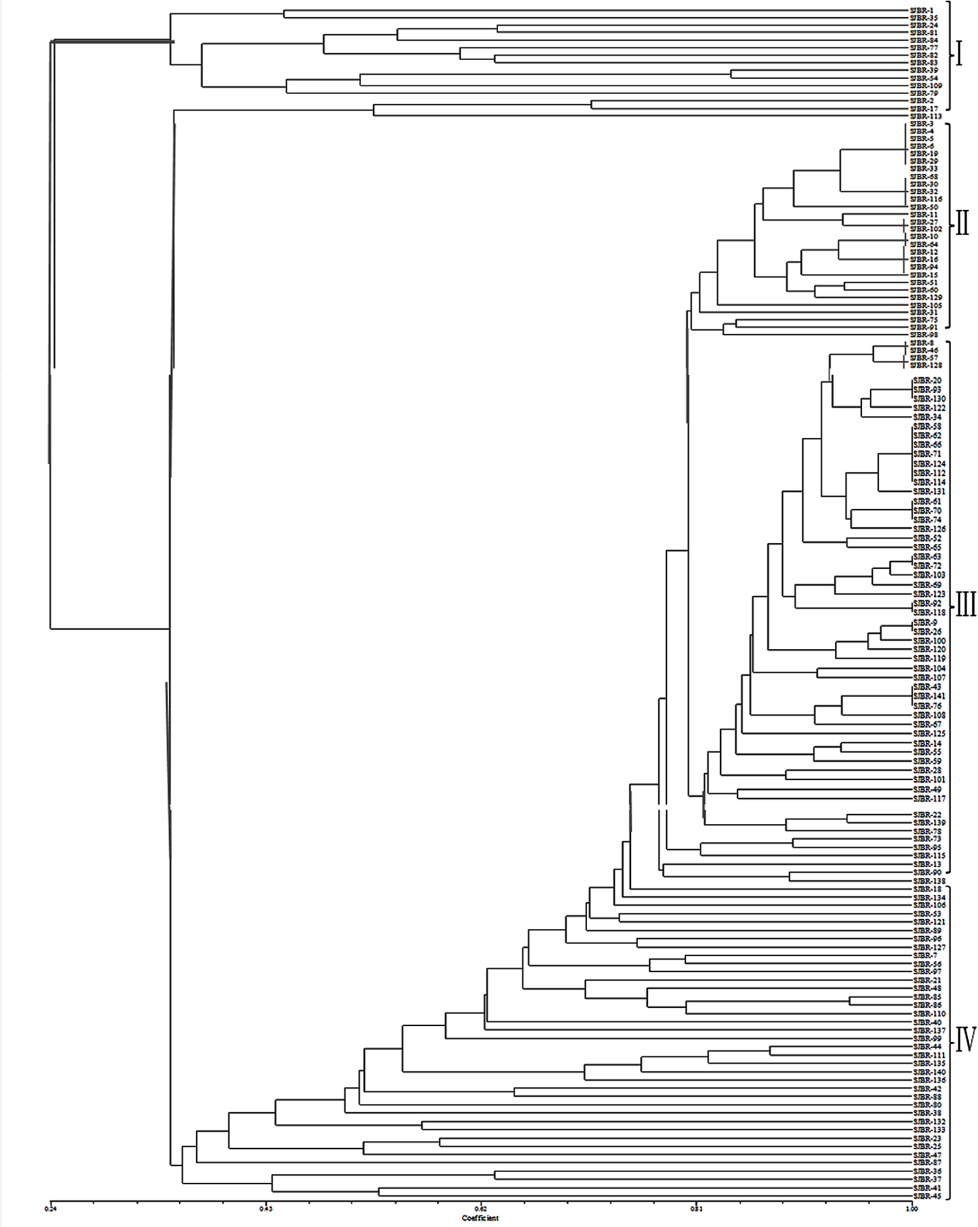
UPGMA dendrogram showing four clusters (I, II, III and IV) of all 141 basmati genotypes of rice.

To find out the genetic relationship between different basmati rice genotypes, SSR data were used for analysis using NTSYSpc version 2.02e. The genetic similarity coefficients found in the genotype comparison matrix were relatively moderate. The distribution analysis of the 9870 pairwise comparisons (Fig 4) revealed extreme values. Zero indicated different genotypes, and 1 indicated similar genotypes. However, most of the values found between 0.2 and 0.9, with an average of 0.60 among all the 141 accessions used indicating a dissimilarity level among the genotypes. Cluster analysis was performed to further elucidate the relationship among the genotypes and the dendrogram generated through UPGMA analysis have been presented in Fig 5, which grouped all genotypes into four major clusters I, II, III and IV, comprising of 15, 29, 59 and 38 genotypes, respectively. The clustering of the basmati rice genotypes was largely based on the place of collection and geographic region. The accessions collected from Badyal, Chatha, Bishnah, and Hansley Chak were grouped in cluster II. The genotypes from Ko Brahimna Samba, Koul Ramgarh Samba, R. S. Pura, Ramgarh and Sainia were clustered in group III. Similarly, collections from SKUAST-J, Kaul Haryana, GBPUAT, IARI and some of the genotypes from PAU were grouped into the cluster IV. Genotypes from Palampur, Meerut, some assessions from IARI, GBPUAT and most of the genotypes from PAU were grouped in cluster I.

### Population structure

**Fig 6:**
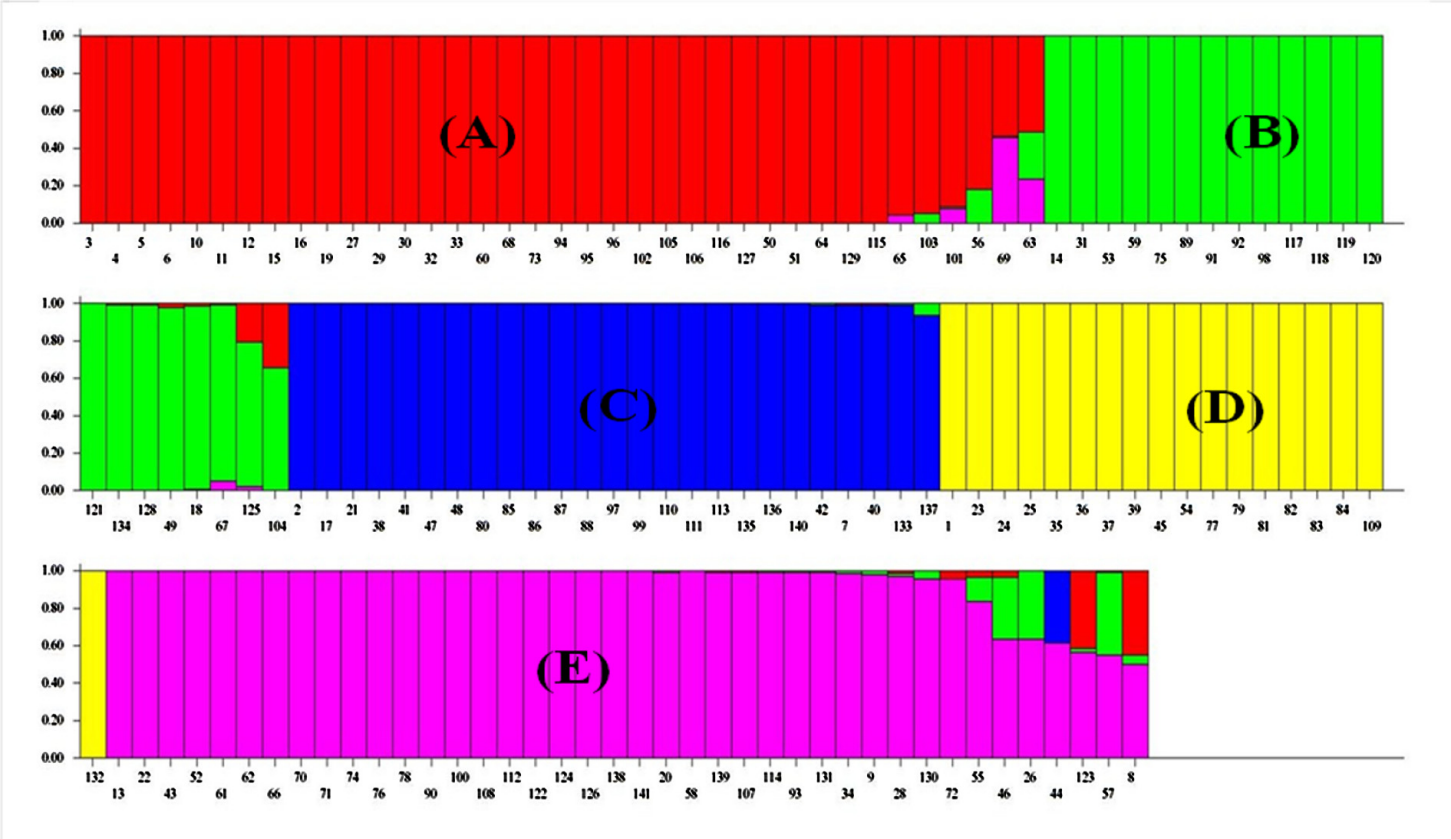
Assignment of 141 basmati rice genotypes to five subpopulations (A, B, C, D and E) using STRUCTURE 2.3.4 software.

**Fig 7:**
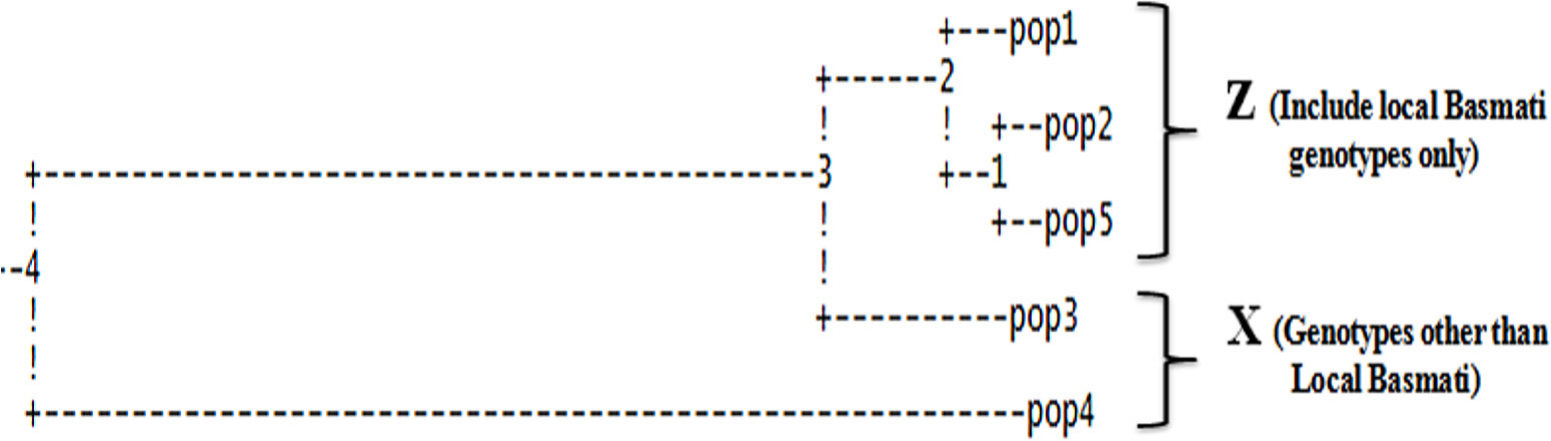
UPGMA dendrogram of five subpopulations. A (pop1), B (pop2), C (pop3), D (pop4) and E (pop5) of basmati rice genotypes showing two clusters Z and X based on Nei’s genetic distances using POPGENE version 1.31.

A model without admixture were carried out by varying K from 2 to 10 with 5 iterations using all 141 genotypes and 38 polymorphic markers. The whole population was stratified into five sub-populations assigned to the corresponding A-E and the inferred population structure are given in Fig 6. The sub-population A, B, C, D and E representing 26.24% (37), 14.89% (21), 17.73% (25), 12.76% (18) and 28.36% (40) of genotypes used in structure analysis respectively. Genetic variation in sub-population was tested using Fst statistics. The sub-populations (A-E) had Fst values of 0.6908, 0.5721, 0.1363, 0.3547 and 0.7470, respectively, with an average value of 0.500 indicating high population structure. Thus, the most structured population was E, followed by A, B, D and C populations. The specific Fst values (not pair-wise Fst values between sub-population) for 5 sub-population (A-E) were calculated using STRUCTURE software during construction of population structure. The average distances (expected heterozygosity) between individuals in same cluster were 0.0681, 0.0960, 0.2608, 0.2925 and 0.0479, respectively. The sub-population A consisted of genotypes collected from Badyal, Chatha, Bishnah, Hansley, Sarore, some accessions from Ko Brahimina, Samba and R. S. Pura. The sub-population B consisted of genotypes collected from Ramgarh and some from R. S. Pura. The sub-population C consisted of genotypes from Kaul, Haryana, some genotypes from PAU, R. S. Pura and majority of SKUAST-J genotypes. The sub-population D consisted of genotypes collected from Palampur, Meerut, GBPUAT, IARI and some genotypes from PAU. The population E consisted of genotypes collected from Koul Ramgarh Samba, Kathua, Sainia, some accession from Ko Brahimina, Samba and R. S. Pura. The dendrogram was also constructed among five subpopulation generated through structure analysis using POPGENE version 1.31 to know relationship among them. The five population were grouped into two clusters (Z and X, respectively), population A (pop1), B (pop2) and E (pop5) were grouped in cluster Z, and population C (pop3) and D (pop4) in cluster X. All the local basmati genotypes were grouped in cluster Z and the genotypes other than local basmati were grouped in cluster X (Fig 7).

### Analysis of molecular variance

The five populations generated from structural analysis were also subjected to analysis of variance (AMOVA) to estimate the percentage of variation among populations and within population. In the total genetic variance among populations based on structure, 39.40% was attributed to the populations based on structure, and the remaining 60.60% was explained by individual differences within populations (Table 3). Pairwise Fst values showed significant differentiation among all the pairs of sub-population ranging from 0.0756 to 0.6873 suggesting that all the five groups were significantly different from each other (Table 4). The sub-population D and E were more differentiated from each other as per the Fst estimate (Table 4).

**Table 3:** Analysis of molecular variance (AMOVA).

Source of variation	d. f.	Sum of square	Variance components	Percentage of variation
Among population	4	150.508	1.29483	39.40
Within population	136	421.369	1.99163	60.60
Total	140	421.369	3.28645	

**Table 4:** Pairwise population differentiation according to groups of populations as measured by Fst using Arlequin.

Populations	A	B	C	D	E
A					
B	0.28725**				
C	0.23277**	0.07565**			
D	0.60642**	0.49270**	0.26014**		
E	0.54412**	0.08732**	0.26775**	0.68732**	

*Significance at P<0.05 at 1,000 permutations

**Significance at P<0.01 at 1,000 permutations

In summary, the results of AMOVA and Fst analysis were in good agreement with the results obtained through phylogenetic tree-based, similarity coefficient distribution and stucture analysis, and confirmed the presence of statistically moderate genetic diversity and high population structure. A critical and important factor to consider before carrying out association mapping (AM) analysis.

## Discussion

The genetic improvement of yield and other economically important traits in crop species depends upon the genetic diversity available within the crop species. The cultivated varieties of basmati rice arise as a result of human selection from the available genetic diversity in various environments and human cultures. Modern breeding in the last two centuries has resulted in the development of varieties that are more uniform, less stable and more adapted to better control and limited environments. This has resulted in the popularization of few genotypes among the farmers, including basmati rice leading to narrow genetic base. The crop had become more prone to biotic and abiotic stresses. The basmati rice improvement requires the identification of highly diverse germplasm and highly polymorphic molecular markers which in turn can be effectively utilized for the mapping of genes/QTLs for economically important traits and their subsequent use in molecular breeding. Hence the present study is initiated to know the genetic base of the basmati germplasm commonly grown in north western Himalayas. Identification of diverse genotypes using molecular markers is advantageous over the conventional approach [39]. SSRs molecular markers have been widely applied in the genetic diversity analysis, genotypic identification and population structure estimation in several rice genetic studies, including basmati rice [1,4–11,40–45]. In the present study, 38 out of 40 markers were polymorphic and produced unique allelic profiles for the 141 basmati rice genotypes. In total 112 alleles were detected among 141 rice genotypes with an average number of 3 alleles per locus and an average polymorphism information content (PIC) of 0.41. The genetic diversity observed in the present study is similar to earlier studies [1], they detected 4.8 alleles per locus and an average PIC value of 0.50. Three alleles per locus with an average PIC value of 0.41 among 88 Indian rice varieties collected from different agro-climatic regions of India were also reported [9]. Similarly, the average PIC value of 0.44 was observed among 43 Thai and 57 IRRI germplasm of rice [46]. In another study, an average PIC value of 0.45 was observed among the 183 Indonesian rice landraces on the Islands of Borneo [47]. A slightly lower genetic diversity was reported with an average of 2.75 alleles per locus and average PIC value of 0.38 among 40 rice accessions of Pakistan [8]. Similarly, a lower SSR diversity was also observed in a study with 36 polymorphic HvSSRs in which they detected 2.22 alleles per locus and an average PIC value of 0.25 in 375 Indian rice varieties collected from different regions of India [7].

In the present study, the average genetic similarity (GS) was observed (0.60) which mostly ranged between 0.2 and 0.9 (Fig 4), reflecting moderate degree of genetic diversity among the genotypes used in this study. The levels of average GS observed in this study, which is also comparable to earlier study [1] in which an average GS of 0.55 was reported among 82 accessions including both Indian and exotic rice was reported. The genetic similarities (GS) ranging from 0.21 to 0.92 among 155 *japonica* rice accessions was also observed [19]. Similarly, an average GS of 0.59 among 88 rice accessions that included landraces, farmer’s varieties and popular basmati lines from India using 50 SSR markers was also reported [9]. The lower average genetic similarities (GS) of 0.39 was observed among 40 elite basmati and non-basmati rice accessions of Pakistan [8]. This is because of less number of diverse germplasm lines of rice have been used for the diversity study.

The dendrogram showed that all 141 genotypes of basmati rice were grouped into four major clusters (Fig 5). The genotypes were well clustered based on their place of collection and geographical region (Figs 5 and 6). The genotypes from Ko Brahimna Samba, Koul Ramgarh Samba, R. S. Pura, Ramgarh, Sainia were grouped in cluster III. Similarly, the genotypes from Badyal, Chatha, Bishnah and Hansley Chak were clustered in cluster II. Thus, most of the local basmati genotypes were clustered in cluster II and cluster III suggesting moderately less genetic diversity among these genotypes. It is because of similar breeding material were used for the development of these genotypes or in other words they have same ancestry. However, the varieties from SKUAST-J, IARI, Kathua, Kaul Haryana, GBPUAT and some from PAU were grouped in cluster IV. The varieties from Palampur, Meerut, some from IARI, GBPUAT and most of the genotypes from PAU were grouped in cluster I. Hence the varieties from IARI, GBPUAT and PAU were present in both cluster I and cluster IV which were distant in dendrogram. This is because of different types of material have been used for the breeding of these varieties.

The population structure analysis revealed 5 subpopulations A, B, C, D and E. The grouping of the genotypes here also is well based on the place of collection and geographic region. The local basmati genotypes were grouped in three subpopulations A, B and E. The genotypes from Badyal, Chatha, Bishnah, Hansley Chak, Sarore, some from Ko Brahimna Samba and R. S. Pura were grouped in sub-population A. Similarly, the genotypes from Koul Ramgarh Samba, Kathua, Sainia, some from Ko Brahimna Samba and R. S. Pura were grouped in subpopulation E. Genotypes from Ramgarh and some from R. S. Pura were grouped in subpopulation B. The varieties from SKUAST-J, Kaul Haryana, some from PAU were grouped in subpopulation C. The varieties from Palampur, Meerut, GBPUAT, IARI and few from PAU were clustered in subpopulation D. Additionally, the presence of statistically significant population structure was confirmed by AMOVA and Fst analyses. These findings are in accordance with earlier studies in which the variation among groups (35.28%) and within groups (64.72%) with a pair-wise Fst estimate ranged from 0.204 and 0.680 [46]. Similarly, the variation among population (34%) and within population (66%) has also been reported [25]. The results obtained through structure analysis and distance-based clustering are in well agreement with each other except the local basmati rice which were clustered into three subpopulations in structure analysis comparison to two cluster in distance-based clustering.

From the similarity coefficient distribution, dendrogram, structure, AMOVA and Fst analysis it is evident that the studied of NW Himalayas basmati rice germplasm has moderate diverse genetic base and high population structure. Hence the most divergent genotypes obtained in this study can be utilized for the future basmati rice breeding programme. Also the diverse genotypes and highly polymorphic functional SSR markers identified during this study can be used for the mapping of QTLs/genes for different biotic and abiotic stresses as well as for quality traits of basmati rice. The present studied basmati germplasm can also be effectively utilized in association mapping (AM) analysis for grain quality traits as is evident from there population structure analysis which is our future objective.

## Supporting Information

S1 Text Availability of germplasm for research purposes (DOC). (DOC)

S2 Text Detailed information about the availability of material (PDF). (PDF)
