# Supplementary material for: Genetic Diversity and Population Structure of Basmati Rice (Oryza sativa L.) Germplasm Collected from North Western Himalayas Using Trait Linked SSR Markers
Source: PLoS One. 2015 Jul 28;10(7):e0131858. doi: 10.1371/journal.pone.0131858 (PMC4517777; doi:10.1371/journal.pone.0131858)
Supplement: S1 Text — (DOC) [file pone.0131858.s001.doc]

**S1. Availability of germplasm for research purposes:** The collected germplasm can be available for research purposes through National Bureau of Plant Genetic Resources and National Biodiversity Authority of India.
