# Supplementary material for: Genetic Diversity and Population Structure of Basmati Rice (Oryza sativa L.) Germplasm Collected from North Western Himalayas Using Trait Linked SSR Markers
Source: PLoS One. 2015 Jul 28;10(7):e0131858. doi: 10.1371/journal.pone.0131858 (PMC4517777; doi:10.1371/journal.pone.0131858)
Supplement: S2 Text — (PDF) [file pone.0131858.s002.pdf]

रजिस्ट्री सं० डी० एल०-33004/99 REGD. NO. D. L.-33004/99

भारत का राजपत्र
The Gazette of India

असाधारण
EXTRAORDINARY
भाग II—खण्ड 3—उप-खण्ड (i)
PART II—Section 3—Sub-section (i)
प्राधिकार से प्रकाशित
PUBLISHED BY AUTHORITY

सं. 612] नई दिल्ली, शुक्रवार, नवम्बर 21, 2014/कार्तिक 30, 1936
No. 612] NEW DELHI, FRIDAY, NOVEMBER 21, 2014/KARTIKA 30, 1936

पर्यावरण, वन और जलवायु परिवर्तन मंत्रालय

(राष्ट्रीय जैव विविधता प्राधिकरण)

अधिसूचना

नई दिल्ली, 21 नवम्बर, 2014

सा.का.नि. 827(अ).—राष्ट्रीय जैव विविधता प्राधिकरण, जैव विविधता अधिनियम, 2002 (2003 का 18) की धारा 18 की उपधारा (1) और धारा 21 की उपधारा (4) के साथ पठित धारा 64 द्वारा प्रदत्त शक्तियों का प्रयोग करते हुए और आनुवांशिक संसाधनों पर पहुंच और उनके उपयोग से उद्भूत फायदों का ऋजु और साम्यपूर्ण विभाजन करने पर नागोया प्रोटोकॉल और जैव विविधता कन्वेंशन तारीख 29 अक्तूबर, 2010 के अनुसरण में निम्नलिखित विनियम बनाता है, अर्थात् :—

संक्षिप्त नाम और प्रारंभ.—(1) इन नियमों का संक्षिप्त नाम जैविक संसाधनों तक पहुंच और सहयुक्त जानकारी तथा फायदा बंटाना विनियम, 2014 है ।

(2) ये राजपत्र में उनके अंतिम प्रकाशन की तारीख से प्रवृत्त होंगे ।

1. जैविक संसाधनों और /या सहयुक्त पारंपरिक जानकारी तक अनुसंधान या जैव सर्वेक्षण तक पहुंच की प्रक्रिया और अनुसंधान के लिए जैव उपयोग :—

(1) अधिनियम की धारा 3 की उपधारा (2) के अधीन निर्दिष्ट कोई व्यक्ति जो जैविक संसाधनों और/या सहयुक्त पारंपरिक जानकारी तक अनुसंधान या जैव सर्वेक्षण तक पहुंच की प्रक्रिया और अनुसंधान के लिए जैव उपयोग का आशय रखता है, राष्ट्रीय जैव विविधता प्राधिकरण (एनबीए) को जैव विविधता नियम, 2004 के प्ररूप-1 में भारत में उपगत होने वाले ऐसे से जैविक संसाधनों और/या सहयुक्त जानकारी तक पहुंच अभिप्राप्त करने के लिए आवेदन करेगा ।

(2) एनबीए उप-विनियम (1) के अधीन किसी आवेदन पर उसका समाधान हो जाने पर, आवेदक के साथ फायदा बंटाने का करार करेगा जिसे उस विनियम में निर्दिष्ट अनुसंधान के लिए अनुमोदन प्रदान करना समझा जाएगा:

परंतु जैविक संसाधनों की दशा में जिनका उच्च स्थानिक मूल्य है, तो करार फायदे को बंटाने के विषय में आवेदक द्वारा एक ऐसी रकम के अग्रिम संदाय के प्रभाव का खंड अंतर्विष्ट हो सकेगा, जो एनबीए और आवेदक के बीच सहमत हो ।

2. वाणिज्यिक उपयोग या जैव सर्वेक्षण के लिए जैविक संसाधनों तक पहुंच की प्रक्रिया और वाणिज्यिक उपयोग के लिए जैव उपयोग-

(1) कोई व्यक्ति जो जैविक संसाधनों तक पहुंच का आशय रखता है, जिसके अंतर्गत संयुक्त वन प्रबंधन समिति (जेएफएमसी)/वनवासी/जनजाति कृषक/ ग्राम सभा द्वारा की जा रही फसल भी है, इन विनियमों से उपाबद्ध प्ररूप 'क' के साथ यथास्थिति जैव विविधता नियम, 2004 के प्ररूप 1 में एनबीए को या राज्य जैव विविधता बोर्ड (एसबीबी) को ऐसे प्ररूप में आवेदन करेगा जो एसबीबी द्वारा विहित किया जाए ।

(2) यथास्थिति, एनबीए या एसबीबी उप विनियम (1) के अधीन आवेदन से समाधान हो जाने पर, आवेदक के साथ फायदा बंटाने का करार करेगा जिसे जैविक संसाधनों के वाणिज्यिक उपयोग के लिए या जैव सर्वेक्षण के लिए और उस उप-विनियम में निर्दिष्ट वाणिज्यिक उपयोग हेतु जैव उपयोग के लिए अनुमोदन समझा जाएगा ।

3. वाणिज्यिक उपयोग के लिए या जैव सर्वेक्षण के लिए और वाणिज्यिक उपयोग हेतु जैव उपयोग के लिए जैविक संसाधनों तक पहुंच के लिए फायदा बंटाने का ढंग

(1) जहां आवेदक/व्यापारी/विनिर्माता ने संयुक्त वन प्रबंधन समिति (जेएफएमसी)/वनवासी/जनजाति कृषक/ग्राम सभा जैसे व्यक्तियों के साथ फायदा बंटाने के लिए पूर्व बातचीत नहीं की है और इन व्यक्तियों से सीधे जैविक संसाधन का क्रय करता है तो व्यापारी पर फायदा बंटाने की बाध्यता जैविक संसाधनों के क्रय मूल्य के 1.0 प्रतिशत से 3.0 प्रतिशत की रेंज में होगी और विनिर्माता पर फायदा बंटाने की बाध्यता जैविक संसाधनों के क्रय मूल्य के 3.0 प्रतिशत से 5.0 प्रतिशत की रेंज में होगी:

परंतु व्यापारी द्वारा उसके द्वारा क्रय किए गए जैविक संसाधनों को किसी अन्य व्यापारी या विनिर्माता को विक्रय करने की दशा में, क्रेता पर फायदा बंटाने की बाध्यता यदि वह व्यापारी है तो क्रय मूल्य के 1.0 प्रतिशत से 3.0 प्रतिशत की रेंज में और यदि वह विनिर्माता है तो 3.0 प्रतिशत से 5.0 प्रतिशत की रेंज में होगी:

परंतु यह और कि यदि क्रेता तुरंत पश्चात्वर्ती विक्रेता के साथ आपूर्ति श्रृंखला में फायदा बंटाने का साक्ष्य प्रस्तुत करता है तो क्रेता पर फायदा बंटाने की बाध्यता केवल उस क्रय मूल्य के भाग तक लागू होगी, जिसके लिए फायदे को आपूर्ति श्रृंखला में बांटा नहीं गया है ।

(2) जहां आवेदक/व्यापारी/विनिर्माता ने संयुक्त वन प्रबंधन समिति (जेएफएमसी) /वनवासी/ जनजाति कृषक/ ग्राम सभा जैसे व्यक्तियों के साथ पूर्व फायदा बंटाने के लिए बातचीत की है और इन व्यक्तियों से सीधे जैविक संसाधन का क्रय करता है तो आवेदक पर फायदा बंटाने की बाध्यता उस दशा में जब क्रेता कोई व्यापारी है, जैविक संसाधनों के क्रय मूल्य के 3.0 प्रतिशत से कम नहीं होगी और क्रेता के विनिर्माता होने की दशा में ये 5.0 प्रतिशत से कम नहीं होगी ।

(3) ऐसे जैविक संसाधनों की दशा में जिनका उच्च आर्थिक मूल्य है जैसे चंदन, लाल सैंडर्स आदि या उनके व्युत्पन्नों की दशा में, फायदा बंटाने में नीलामी या विक्रय की रकम से आगतों के 5 प्रतिशत से अन्यून का संदाय जैसा कि, यथास्थिति, एनबीए या एसबीबी द्वारा विनिश्चय किया जाए और सफल बोली दाता या क्रय कर्ता अभिहित निधि में जैविक संसाधनों तक पहुंच से पूर्व रकम का संदाय करेगा ।

4. विनियम 2 के अधीन वाणिज्यिक उपयोग के लिए पहुंच बनाए गए जैविक संसाधनों के विक्रय मूल्य के फायदे को बंटाने का विकल्प-

जब जैविक संसाधनों तक वाणिज्यिक उपयोग के लिए या जैव सर्वेक्षण के लिए पहुंच की जाती है और जैव उपयोग से वाणिज्यिक उपयोग होता है तो आवेदक के पास फायदा बंटाने का 0.1 प्रतिशत से 0.5 प्रतिशत की रेंज में उस उत्पाद की वार्षिक समग्र कारखाना पूर्व विक्रय की प्रतिशतता के निम्नलिखित ग्रेड में जिसकी गणना वार्षिक कारखाना पूर्व समग्र बिक्री को सरकारी करों से कम करके, जैसा कि नीचे दिया गया है, विकल्प होगा

**उत्पाद का कारखाना पूर्व वार्षिक विक्रय**	**फायदा बंटाने वाले संघटक**
1,00,00,000 रुपए तक	0.1 %
1,00,00,001 रुपए से 3,00,00,000 रुपए तक	0.2%
3,00,00,000 रुपए से ऊपर	0.5%

5. फीस का संग्रहण—

अधिनियम की धारा 41 की उपधारा (3) के अधीन किसी जैविक संसाधन तक वाणिज्यिक प्रयोजन के लिए पहुंच या संग्रहण के लिए उन क्षेत्रों से जो जैव विविधता प्रबंधन समिति (बीएमसी) के राज्यक्षेत्रीय क्षेत्राधिकार में आते हैं, फीस का संग्रहण इन विनियमों के अधीन एनबीए/एसबीबी को फायदा बंटाने के अतिरिक्त होगा ।

6. जैविक संसाधनों से संबंधित अनुसंधान के परिणामों को अंतरित करने की प्रक्रिया—

(1) धनीय प्रतिफल या अन्यथा के लिए अधिनियम की धारा 3 की उप-धारा (2) में निर्दिष्ट व्यक्तियों को भारत में उपगत होने वाले या भारत में अभिप्राप्त जैविक संसाधनों से संबंधित अनुसंधान के परिणामों को अंतरित करने का आशय रखने वाला कोई व्यक्ति—

(क) भारत में अभिप्राप्त जैविक संसाधनों से संबंधित अनुसंधान के परिणामों को किसी प्रयोजन के लिए अंतरित करने के लिए जैविक विविधता नियम, 2004 के प्ररूप 2 में आवेदन करेगा ;

(ख) अनुसंधान में अंतवर्लित जैविक संसाधन और / या सहयुक्त जानकारी तक पहुंच के लिए एनबीए के अनुमोदन का साक्ष्य प्रस्तुत करेगा:

परंतु किसी आवेदक को, जो भारत का नागरिक है या निगमित निकाय, संगम या संगठन है जो भारत में रजिस्ट्रीकृत है और उसकी पूंजी या प्रबंधन में किसी गैर भारतीय की कोई सहभागिता नहीं है, इस खंड के अधीन साक्ष्य देने की अपेक्षा लागू नहीं होगी ;

(ग) आवेदक की जानकारी में अनुसंधान के परिणामों के संभावित वाणिज्यिक मूल्य की संपूर्ण जानकारी देगा ।

(2) एनबीए, उप-विनियम (1) के अधीन आवेदन से समाधान हो जाने पर, आवेदक के साथ एक फायदा बंटाने का करार करेगा जिसे उस विनियम में निर्दिष्ट अनुसंधान के परिणामों को अंतरित करने का अनुमोदन समझा जाएगा ।

7. अनुसंधान के परिणामों को अंतरित करने के लिए फायदे बंटाने का ढंग-आवेदक, विनियम 6 के अधीन अनुसंधान के परिणामों को अंतरित करने की दशा में एनबीए को ऐसे धनीय और गैर धनीय फायदे का संदाय करेगा जैसी कि आवेदक और एनबीए के बीच सहमति हुई हो :

परंतु उसके द्वारा धनीय फायदे की प्राप्ति पर, यदि कोई हो, ऐसे अंतरण पर आवेदक एनबीए को धनीय विचारण का 3.0 प्रतिशत से 5.0 प्रतिशत तक का संदाय करेगा ।

8. बौद्धिक संपदा अधिकार (आईपीआर) अभिप्राप्त करने की प्रक्रिया—

(1) कोई व्यक्ति जो किसी बौद्धिक संपदा अधिकार को भारत या भारत से बाहर अभिप्राप्त करने का आशय रखता है, चाहे किसी भी नाम से ज्ञात हो, किसी खोज के लिए जो किसी भारत में अभिप्राप्त अनुसंधान या किसी जैविक संसाधन पर किसी सूचना पर आधारित है, जैविक विविधता नियम, 2004 के प्ररूप 3 में एनबीए को आवेदन करेगा:

परंतु यदि आवेदक अधिनियम की धारा 3 की उप-धारा (2) में निर्दिष्ट कोई व्यक्ति है, तो वह जैविक संसाधनों और/या सहयुक्त जानकारी के उपयोग, जो उसके अनुसंधान में उपयोग की गई है जिससे वह खोज हुई है, तक पहुंच के लिए एनबीए के अनुमोदन का साक्ष्य प्रस्तुत करेगा:

परंतु यह और कि पौधा किस्म और कृषक अधिकार अधिनियम, 2001 (2001 का 53) के अधीन किसी अधिकार के लिए आवेदन करने वाले किसी व्यक्ति को इस उप-विनियम से छूट होगी ।

(2) एनबीए उप विनियम (1) के अधीन आवेदन की प्राप्ति पर समाधान हो जाने पर आवेदक के साथ फायदा बंटाने का करार करेगा और इसे आईपीआर अभिप्राप्त करने का अनुमोदन माना जाएगा ।

9. आईपीआर में हिस्सा बंटाने का ढंग-

(1) आवेदक, अभिप्राप्त आईपीआर के वाणिज्यिकरण की दशा में एनबीए को ऐसा धनीय और/या गैर धनीय फायदे का संदाय करेगा जैसे कि आवेदक और एनबीए के बीच सहमति हो ।

(2) जहां आवेदक स्वयं प्रकिया/उत्पाद /नई पद्धति का वाणिज्यिकरण करता है, धन सेक्ट्रल अप्रोच के आधार पर जिसकी गणना सरकारी करों को घटाकर वार्षिक समग्र कारखाना पूर्व विक्रय के आधार पर की जाएगी, 0.2 से 1.0 प्रतिशत की रेंज में बांटा जाएगा ।

(3) जहां आवेदक प्रकिया को अनुज्ञप्त/उत्पाद/नई पद्धति को वाणिज्यिकरण के लिए तृतीय पक्षकार को समानुदेशित करता है, (किसी भी रूप में जिसके अंतर्गत अनुज्ञप्ति/समनुदेशन फीस है) आवेदक एनबीए को प्राप्त फीस का 3.0 प्रतिशत से 5.0 प्रतिशत और सेकटोरल अप्रोच के आधार पर समानुदेशिति/अनुज्ञप्तिधारी से वार्षिक रूप से प्राप्त स्वामिस्व की रकम का 2.0 प्रतिशत से 5.0 प्रतिशत के बीच संदाय करेगा ।

10. आईपीआर के वाणिज्यिकरण की दशा में आवेदक की बाध्यताएं-

(1) कोई आवेदक, जिसे आईपीआर प्रदान किया गया है, जो भारत का नागरिक है या निगमित निकाय है, संगम या संगठन है जो भारत में रजिस्ट्रीकृत है और जिसकी शेयर पूंजी या प्रबंधन में किसी गैर भारतीय की सहभागिता नहीं है जैविक संसाधनों तक पहुंच के लिए संबंधित एसबीबी को एसबीबी द्वारा विहित प्ररूप में पूर्व सूचना देगा और ऐसे निबंधनों और शर्तों का अनुपालन करेगा, यदि कोई हों, जो एसएसबी द्वारा संरक्षण और धारणीय उपयोग के हित में अधिरोपित की जाएं ।

(2) कोई आवेदक जो अधिनियम की धारा 3 की उपधारा (2) में निर्दिष्ट व्यक्ति है, जिसे आईपीआर प्रदान किया गया है और जो निगमित निकाय या कोई संगम या कोई संगठन है, जैविक विविधता नियम, 2004 के प्ररूप 1 में एनबीए को जैविक संसाधनों तक पहुंच के लिए आवेदन करेगा ।

11. ऐसे जैविक संसाधनों जिन तक पहुंच की गई है और /या सहयुक्त जानकारी को अनुसंधान के लिए /वाणिज्यिक उपयोग के लिए तृतीय पक्षकार को अंतरित करने की प्रक्रिया-

(1) कोई व्यक्ति जो, जैविक संसाधनों को और /या सहयुक्त जानकारी को अंतरित करने का आशय रखता है जिसे विनियम 1 के अधीन तृतीय पक्षकार को अनुसंधान या वाणिज्यिक उपयोग के लिए पहुंच प्रदान की गई है, जैविक विविधता नियम, 2004 के प्ररूप 4 में एनबीए को ऐसे तृतीय पक्षकार को अंतरण के लिए आवेदन करेगा ।

(2) एनबीए उप विनियम (1) के अधीन आवेदन से समाधान हो जाने पर आवेदक हिस्सा बंटाने का करार करेगा जिसे पहुंच किए गए जैविक संसाधनों और /या उस विनियम में निर्दिष्ट सहयुक्त जानकारी के अंतरण के लिए अनुमोदन प्रदान करना समझा जाएगा ।

12. जैविक संसाधनों जिन तक पहुंच की गई है और या सहयुक्त जानकारी को अनुसंधान/वाणिज्यिक उपयोग के लिए तीसरे पक्षकार को अंतरित करने का ढंग-

(1) आवेदक एनबीए को ऐसे धन और या गैर धनीय फायदे को संदाय करेगा जैसा कि आवेदक और एनबीए के बीच सहमति हो ।

(2) आवेदक (अंतरक) एनबीए को किसी रकम और/या अंतरिती से प्राप्त राजस्व का करार की संपूर्ण अवधि के दौरान फायदा बंटाने के रूप में 2.0 प्रतिशत से 5.0 प्रतिशत (सेक्ट्रल अप्रोच का अनुसरण करते हुए) का संदाय करेगा ।

(3) जैविक संसाधन का उच्च आर्थिक मूल्य होने की दशा में, आवेदक, एनबीए को एक अग्रिम संदाय का भी जैसा कि आवेदक और एनबीए के बीच सहमति हो, संदाय करेगा ।

13. भारतीय अनुसंधानकर्ताओं/संस्थाओं द्वारा भारत से बाहर जैव वाणिज्यिक अनुसंधान करना या आपातकालीन प्रयोजनों के लिए अनुसंधान करना :-

(1) कोई भारतीय अनुसंधानकर्ता/सरकारी संस्था जो जैविक संसाधनों को अधिनियम की धारा 5 में निर्दिष्ट सहयोगी अनुसंधान से भिन्न आधारभूत अनुसंधान करने के लिए ले जाने/भेजने का आशय रखता है, एनबीए को इन विनियमों से उपाबद्ध प्ररूप 'ख' में आवेदन करेगा ।

(2) कोई सरकारी संस्था जो महामारी आदि से बचने के लिए कतिपय शीघ्र अध्ययन करने के लिए जैविक संसाधन भेजने का आशय रखता है, एनबीए को इन विनियमों से उपाबद्ध प्ररूप 'ख' में आवेदन करेगा ।

(3) एनबीए उप विनियम (1) या उप विनियम (2) के अधीन आवेदन से समाधान हो जाने पर, आवेदन की प्राप्ति की तारीख से पैंतालीस दिन के भीतर अपना अनुमोदन प्रदान करेगा ।

(4) उप विनियम (3) के अधीन एनबीए से अनुमोदन की प्राप्ति पर, आवेदक भारत से बाहर जैविक संसाधनों को ले जाने /भेजने से पूर्व अभिहित राष्ट्रीय निक्षेपागार को वाउचर नमूने जमा करेगा और ऐसे जमा की प्रति सबूत के रूप में एनबीए को पृष्ठांकित किया जाएगा ।

14. फायदा बंटाने का अवधारण-

(1) धनीय और गैर धनीय ढंग से फायदा बंटाना जैसी कि आवेदक और एनबीए /संबंधित एसबीबी के बीच सहमति हो, बीएमसी/फायदे का दावा करने वाले आदि के बीच परामर्श से किया जा सकेगा । ऐसे फायदा बंटाने के लिए विकल्प उपाबंध 1 में दिए गए हैं ।

(2) फायदा बंटाने का अवधारण विचारणों पर आधारित होगा जैसे कि जैविक संसाधन का वाणिज्यिक उपयोग, अनुसंधान और विकास के प्रक्रम, अनुसंधान के परिणाम के लिए संभावित बाजार अनुसंधान और विकास में पहले ही किए गए विनिधान की रकम उपयोग की गई प्रौद्योगिकी की प्रकृति अनुसंधान प्रारंभ करने से उत्पाद के विकास तक समय और प्राप्त की गई उल्लेखनीय उपलब्धियां तथा उत्पाद के वाणिज्यिकरण में अंतवर्लित जोखिम:

परंतु ऐसे मामलों पर विशेष ध्यान दिया जा सकेगा जहां प्रौद्योगिकियों/उत्पादों का विकास महामारियों/बीमारियों को नियंत्रित करने के लिए और मानव /प्राणी/पादप स्वास्थ्य को प्रभावित करने वाले पर्यावरणीय प्रदूषण को कम करने के लिए किया जाता है ।

(3) फायदा बंटाने की रकम समान रहेगी चाहे अंतिम उत्पाद में एक या अधिक जैविक संसाधन अतर्विष्ट हैं ।

(4) किसी उत्पाद के लिए, दो या अधिक एसबीबी के क्षेत्राधिकार से प्राप्त जैविक संसाधनों की दशा में उद्‌भूत फायदों की कुल रकम को उनकी बीच एनबीए द्वारा यथा विनिश्चित समानुपात में बांटा जाएगा ।

15. फायदों का बंटाना-

(1) जहां एनबीए द्वारा अनुमोदन अनुसंधान या वाणिज्यिक उपयोग या अनुसंधान के परिणाम के अंतरण के लिए या बौद्धिक संपदा अधिकारों या तृतीय पक्षकार अंतरण के लिए प्रदान किया जाता है, हिस्सा बंटाने का ढंग नीचे दिए अनुसार होगा-

(क) उद्‌भूत फायदों 5.0 प्रतिशत एनबीए को मिलेगा जिसमें से आधी रकम को एनबीए द्वारा प्रतिधारित किया जाएगा और शेष आधी रकम को प्रशासनिक प्रभारों के लिए संबंधित एसबीबी को दिया जा सकेगा ।

(ख) उद्‌भूत फायदों का 95 प्रतिशत संबंधित बीएमसी को और/या फायदे का दावा करने वालों को मिलेगा :

परंतु जैविक संसाधन या जानकारी किसी व्यष्टि या व्यष्टियों के समूह या संगठन से प्राप्त करने की दशा में, इस खंड के अधीन रकम सीधे ऐसे व्यष्टि या व्यष्टियों के समूह या संगठनों को किसी करार के निबंधनों के अनुसार और ऐसी रीति में जो ठीक समझी जाए, दी जाएगी:

परंतु यह और कि जहां फायदे का दावा करने वालों की पहचान नहीं की गई है, ऐसी निधि का उपयोग जैविक संसाधनों के संरक्षण और धारणीयता का उपयोग करने के लिए और उन स्थानीय व्यक्तियों के जीवन यापन का संबर्द्धन करने के लिए जहां संसाधन प्राप्त किए गए हैं, किया जाएगा ।

(2) जहां अनुमोदन इन विनियमों के अधीन राज्य जैव विविधता बोर्ड द्वारा प्रदान किया गया है ।एसबीबी उद्‌भूत फायदों का 5 प्रतिशत से अनधिक भाग का प्रशासनिक प्रभारों के लिए प्रतिधारण कर सकेगा और शेष हिस्सा संबधित बीएमसी या फायदे का दावा करने वालों को, जहां उनकी पहचान की गई है, दे दिया जाएगा:

परंतु यह कि जहां व्यष्टि या व्यष्टियों के समूह या संगठनों की पहचान नहीं की जा सकती है, ऐसी निधियों का उपयोग जैविक संसाधनों के संरक्षण और धारणीयता का उपयोग करने के लिए और उन स्थानीय व्यक्तियों के जीवन यापन का संबर्द्धन करने के लिए जहां संसाधन प्राप्त किए गए हैं, किया जाएगा ।

16. एनबीए द्वारा प्राप्त आवेदनों को प्रक्रियागत् करना-

(1) प्रत्येक आवेदन उसमें निर्दिष्ट संलग्नकों सहित सभी परिप्रेक्ष्यों में पूर्ण होगी ।

(2) अपूर्ण आवेदन जिनमें कोई सूचना जो विशिष्ट रूप से चाही गई है जिसके अंतर्गत अस्पष्ट प्रत्युत्तर, अपूर्ण प्रकटन, साक्ष्य का अभाव आदि हैं, को आवेदकों को लौटा दिया जाएगा ।

(3) आवेदनों को प्रक्रियागत् करने के लिए विनिर्दिष्ट समय सीमा केवल तब प्रारंभ होगी जब आवेदन सभी परिप्रेक्ष्यों में जिसके अंतर्गत विहित फीस का प्राप्त करना है, पूर्ण हों ।

(4) आवेदन में किसी सूचना, जिसे गोपनीय विनिर्दिष्ट किया गया है, का प्रकटन जान बूझकर या अनजाने में किसी व्यक्ति को जो उससे संबंधित नहीं है, नहीं किया जाएगा ।

(5) एनबीए, किसी जैविक संसाधन (जिसे अंतर्गत पादप और /या प्राणी और/या उनके भाग या आनुवांशिक सामग्री या व्युतपन्न हैं) तक पहुंच के लिए किसी आवेदन का प्रसंस्करण करते हुए, निम्नलिखित कारकों पर विचार कर सकेगा, अर्थात्:-

क्या जैविक संसाधन की -

(i) कृषि की गई है या पालतु या वन्य है ;

(ii) दुर्लभ या स्थानिक या संकटापन्न या जोखिम में प्रजाति है;

(iii) पहुंच सीधे प्रारंभिक संग्राहक के माध्यम से की गई है जो प्राकृतिक पर्यवास में रह रहा है या उसे मध्यवर्तियों जैसे व्यापारियों के माध्यम से अभिप्राप्त किया गया है ;

(iv) विकास या अनुरक्षण पूर्व के आस-पास की परिस्थितियों के अधीन किया गया है ;

(v) स्थानीय समुदायों के जीवन यापन के लिए उच्च मूल्य/महत्ता है ;

(vi) अधिनियम या तत्समय प्रवृत्त किसी अन्य विधि के अधीन निर्बंधित है ;

(vii) अधिनियम की धारा 40 के अधीन छूट प्राप्त है ;

(viii) अंतरराष्ट्रीय खाद्य और कृषि के लिए पादप अनुवांशिकी संसाधन संधि (आईटीपीजीआरएफए,) जिसमें भारत एक संविदाकारी पक्षकार है, के उपाबंध 1 के अधीन सूचीबद्ध फसलों में शामिल है;

(ix) संकटापन्न प्रजाति का अंतरराष्ट्रीय व्यापार पर कन्वेंशन के परिशिष्ट (सीआईटीईएस) में शामिल है ।

(6) एनबीए, जैविक संसाधनों और /या सहयुक्त जानकारी के उपयोग से संबंधित आवेदन पर कोई विनिश्चय करते समय एसबीबी, बीएमसी, जिनकी अधिकारिता के भीतर जैविक संसाधन और/या जानकारी उद्भूत होती है, से परामर्श कर सकेगा

(7) एनबीए जैविक विविधता नियम, 2004 के नियम 16 के अधीन विनिर्दिष्ट कारणों से जैविक संसाधनों तक पहुंच के आवेदन को अस्वीकार कर सकेगा ।

(8) किसी आवेदन की प्राप्ति पर एनबीए ऐसी जांच कर सकेगा जैसा कि वह ठीक समझे और यदि आवश्यक हो तो वह इस प्रयोजन के लिए गठित विशेषज्ञ समिति से परामर्श कर सकेगा ।

(9) एनबीए, ऐसी जांच और/या उप विनियम (8) में निर्दिष्ट परामर्श करने पर आदेश द्वारा अनुमोदन प्रदान कर सकेगा या आवेदन को अस्वीकार कर सकेगा:

परंतु जहां एनबीए ने ऐसे आवेदन को अस्वीकार कर दिया है, ऐसे अस्वीकार करने के कारणों को आवेदक को सुने जाने का अवसर दिए जाने के पश्चात्, लेखबद्ध किया जाएगा ।

(10) एनबीए द्वारा किया गया अनुमोदन एनबीए को सम्यक् रूप से प्राधिकृत अधिकारी, आवेदक और यथा लागू अन्य के बीच हस्ताक्षरित लिखित करार के रूप में होगा :

परंतु एनबीए गैर वाणिज्यिक अनुसंधान करने या भारत से बाहर भारतीय अनुसंधानकर्ताओं/संस्थाओं द्वारा विनियम 13 के अधीन आपातकालीन प्रयोजनों के लिए अनुसंधान करने के लिए बिना किसी लिखित करार के अनुमोदन प्रदान कर सकेगा ।

(11) एनबीए, किसी शिकायत के आधार पर या स्व प्रेरणा से पहुंच के लिए प्रदान किए गए अनुमोदन को वापस ले सकेगा और जैविक विविधता नियम, 2004 के नियम 15 के अधीन विनिर्दिष्ट आधारों पर लिखित करार का प्रतिसंहरण कर सकेगा:

परंतु ऐसे प्रतिसंहृण के आदेश की प्रति संबंधित राज्य जैव विविधता बोर्ड को और जैव विविधता प्रबंधन समितियों को पहुंच को प्रतिषिद्ध करने के प्रयोजन के लिए जारी की जाएगी ।

(12) जहां आवेदक द्वारा आवेदन को वापस लेने के लिए अनुरोध किया जाता है या आवेदक अनुबद्ध समय के भीतर एनबीए के प्रश्नों का प्रत्युत्तर देने में असफल रहता है, एनबीए आवेदनों को बंद कर देगा या इस अधिनियम के अधीन जैसा वह उचित समझे कार्रवाई प्रारंभ करेगा:

परंतु यह कि यदि आवेदक आवेदन को पुनर्जीवित करना चाहता है तो वह अपेक्षित फीस के साथ नया आवेदन करेगा ।

टिप्पण: जैविक संसाधनों तक पहुंच या सहयुक्त जानकारी, मार्गदर्शक सिद्धांतों के लिए भरने के लिए आवेदन फार्म और करार के फार्म एनबीए की वेबसाईट: www.nbaindia.org पर उपलब्ध हैं ।

17. एनबीए या एसबीबी से छूट प्राप्त कतिपय कार्यकलाप या व्यक्ति-

निम्नलिखित कार्यकलाप या व्यक्तियों को एनबीए या एसबीबी के अनुमोदन की अपेक्षा नहीं होगी, अर्थात् :-

(क) जैविक संसाधनों और /या सहयुक्त जानकारी को, जो भारत में उद्भूत हो रही है या भारत में अभिप्राप्त है, अनुसंधान या जैविक सर्वेक्षण और भारत में अनुसंधान के लिए जैव उपयोग के लिए पहुंच करते हुए भारतीय नागरिक या निकाय;

(ख) सहयोगकारी अनुसंधान परियोजनाएं, जिनमें जैविक संसाधनों या संबंधित जानकारी का अंतरण या विनिमय अंतवर्लित है, यदि ऐसी सहयोगकारी अनुसंधान परियोजनाओं को राज्य या केन्द्रीय सरकार के संबंधित मंत्रालय या विभाग द्वारा अनुमोदित किया गया है और वे ऐसी सहयोगकारी अनुसंधान परियोजनाओं को केन्द्रीय सरकार द्वारा जारी नीति मार्गदर्शक सिद्धांतों के अनुरूप हैं;

(ग) क्षेत्र के स्थानीय लोग और समुदाय, जिनके अंतर्गत जैविक संसाधनों को उगाने वाले और उनकी कृषि करने वाले सिवाय बौद्धिक संपदा अधिकार अभिप्राप्त करने के, तथा स्वदेशी ओषधि का व्यवसाय करने वाले वैद्य और हकीम भी हैं ;

(घ) पारंपरिक प्रजनन या भारत में किसी कृषि, बागवानी, कुक्कुट पालन, दुग्ध उद्योग, पशु पालन या मधु मक्खी पालन के लिए पारंपरिक पद्धतियों का उपयोग;

(ङ) अनुसंधान पेपरों का प्रकाशन या किसी सेमिनार या कार्यशाला में जानकारी का प्रसार यदि ऐसा प्रकाशन केन्द्रीय सरकार द्वारा समय समय पर जारी मार्गदर्शक सिद्धांतों के अनुसार है ;

(च) मूल्यवर्धित उत्पाद, जो ऐसे उत्पाद हैं जिनमें पादपों और प्राणियों का निष्कर्षण पहचानी न जा सकने वाली और भौतिक रूप से अलग न किए जा सकने वाले रूप में है ;

(छ) जैविक संसाधन, जिनका साधारणतया व्यापार केन्द्रीय सरकार द्वारा अधिनियम की धारा 40 के अधीन अधिसूचित सामग्रियों के रूप में होता है ।

[फा. सं. एनबीए/टेक / 2/11]

हेम पाण्डे, अध्यक्ष्

प्ररूप क

(विनियम 2 देखिए)

आवेदक द्वारा जैविक संसाधनों के उपयोग के लिए प्रस्तुत

की जाने वाली सूचना

स्वत: प्रकटन

उपयोग किए जाने के लिए प्रस्तावित जैविक संसाधन का सामान्य नाम:				
वैज्ञानिक नाम:				
व्यापार किए जाने वाले पादप या प्राणी या उनके भाग:				
पहुंच का विशिष्ट प्रयोजन:				
अवस्थिति/स्रोत जहां से उपाप्त किए गए है*	मात्रा किलो ग्राम में	दर प्रति इकाई	राज्य जैविक विविधता बोर्ड	भावी क्रेता/उपयोगकर्ता (यदि ज्ञात हों)

*स्थानीय निकाय/बीएमसी, यदि पहले ही पहचान कर ली गई है तो सूची संलग्न करें

वचनबंध

1. मैंने एबीएस मार्गदर्शक सिद्धांतों के निबंधनों और शर्तों को पढ़ और समझ लिया है तथा मैं जैविक संसाधनों को लागू सुसंगत विधिक उपबंधों का पालन करने का वचन देता हूं ।

2. मैं कथित प्रयोजन में कोई परिवर्तन करने से पूर्व एनबीए/एसबीबी का अनुमोदन अभिप्राप्त करने का वचन देता हूँ ।

3. मैं, एनबीए/एसबीबी को सुसंगत अभिलेख प्रस्तुत करने /दिखाने का, जब भी अपेक्षा हो, वचन देता हूँ ।

4. मैं यह और घोषणा करता हूँ कि प्ररूप में दी गई सूचना सत्य और सही है तथा मैं किसी असत्य /गलत जानकारी और जानबूझकर तथ्यों को छिपाने के लिए उत्तरदायी होउंगा ।

हस्ताक्षर

व्यापारी/कंपनी/विनिर्माता/प्राधिकृत प्रतिनिधि का नाम

व्यापारी/कंपनी/विनिर्माता के पूरे पते के साथ फोन नं. और ई-मेल का पता

स्थान

तारीख

प्ररूप ख

(देखिए विनियम 13)

भारतीय अनुसंधानकर्ताओं/संस्थाओं द्वारा जैविक संसाधनों का उपयोग करते हुए गैर वाणिज्यिक अनुसंधान या आपातकालीन प्रयोजनों के लिए अनुसंधान

1.	आवेदक का नाम (भारतीय अनुसंधानकर्ता/संस्था)	
2.	पूरा पता*	
	क. स्थायी<br>ख. वर्तमान	
3.	भारत में संस्थान का पता	
4.	भारत में कार्य स्थल पर संस्था के पर्यवेक्षक या अध्यक्ष का नाम	
5.	संस्थान या संगठन का नाम और संपर्क के ब्यौरे जो प्रस्तावित अनुसंधान का मार्गदर्शन करेगा /जैविक संसाधनों को प्राप्त करेगा	
6.	संस्थान या संगठन के पर्यवेक्षक के ब्यौरे जो प्रस्तावित अनुसंधान का मार्गदर्शन करेगा /जो जैविक संसाधनों का प्राप्तकर्ता है	
7.	प्रस्तावित अध्ययन की सहायता करने वाले वित्त पोषण अभिकरण का नाम	
8.	अनुसंधान का संक्षिप्त विवरण	
9.	अनुसंधान के लिए साथ ले जाए जाने वाले या भेजे जाने वाले प्रस्तावित जैविक संसाधनों के ब्यौरे	
	i. जैविक संसाधन का नाम (वैज्ञानिक /सामान्य नाम)<br>ii. संग्रहण की अवस्थिति<br>iii. अपेक्षित मात्रा<br>iv. अनुसंधान की अवधि	
10.	यदि ये आपातकालीन प्रयोजन के लिए है तो ब्यौरे विनिर्दिष्ट करें।	

* स्वयं द्वारा हस्ताक्षरित पते /पहचान के सबूत जैसे कि आधार कार्ड/पेन कार्ड/पासपोर्ट आदि संलग्न करें।

वचनबंध

में ----------पुत्र/पुत्री/पत्नी/पति --------------आयु----------- में निवास कर रहा हूँ, मैं स्थायी पहचान सं.-------------- (पेन कार्ड/आधार कार्ड/पासपोर्ट आदि) का धारक हूँ, एतद्वारा घोषणा करता हूँ कि ऊपर दी गई सभी जानकारी सही और सत्य है। मैं एतद् द्वारा पुष्टि करता हूं कि जैविक संसाधनों का उपयोग केवल आवेदन में कथित प्रयोजनों के लिए ही किया जाएगा। मैं किसी सहयोगकर्ता की प्रसुविधा/प्रयोगशाला में एनबीए का अनुमोदन प्राप्त किए बिना किसी जैविक संसाधन को नहीं बांटूंगा/उपलब्ध कराउंगा/छोडूंगा/रहने दूंगा। मैं अपने पर्यवेक्षक और सहयोगकर्ता के साथ व्यष्टिक रूप से और पृथक् रूप से घोषणा करता हूँ कि न तो जैविक संसाधनों और सहयुक्त पारंपरिक जानकारी जिसका उपयोग अनुसंधान/सहयोग में किया गया है, का वाणिज्यिक उपयोग करुंगा न ही उस पर कोई आईपीआर दावा करुंगा। यदि ऐसी स्थिति उत्पन्न होती है तो हम पूर्वानुमोदन प्राप्त करने के लिए राष्ट्रीय जैव विविधता प्राधिकरण को आवेदन करेंगे। इस कार्यकलाप से उद्भूत परिणामों, प्रक्रियाओं, उत्पादों या अन्य नतीजों को हम अनुसंधान के दौरान और अनुसंधान के पूरा होने पर एनबीए के साथ सुसंगत दस्तावेजों और प्रकाशनों की प्रति के साथ बांटेंगे।

हस्ताक्षर--------------------
तारीख----------------------
स्थान-----------------------

संस्था के पर्यवेक्षक/अध्यक्ष द्वारा घोषणा

मैं----------------- के रूप में--------------- में (संस्था का नाम) पुष्टि करता हूँ कि -- श्री/डा./श्रीमती/सुश्री------------------द्वारा दिए गए ब्यौरे सत्य और सही हैं।

तारीख------------<br><br>स्थान---------------	हस्ताक्षर-----------------<br><br>पदनाम--------------------<br><br>कार्यालय की मुहर-------------

प्राप्तकर्ता/सहयोगकर्ता द्वारा घोषणा

मैं -----------------के रूप में ------------------------में कार्यरत (संस्था/संगठन का नाम) पुष्टि करता हूँ कि मैं या मेरी संस्था/संगठन जैविक संसाधनों का उपयोग आवेदन में कथित प्रयोजनों के लिए ही करेंगे और जिसे ---------------द्वारा (संस्था का नाम भेजा गया है) या जिसे श्री/ डा./श्रीमती/सुश्री द्वारा लाया गया है, यथास्थिति, उक्त जैविक संसाधन अध्ययन/भागीदारी के पूरा होने के पश्चात् पूर्णतया नष्ट कर दिए जाएंगे या अध्ययन के पश्चात् जैविक संसाधनों को उस संस्था को जहां से जैविक संसाधन प्राप्त किए गए थे को वापस भेज दिया जाएगा । मैं या संस्था जिसके साथ में सहयुक्त हूँ त्वरित आवेदन के अधीन किसी स्वामित्व का कोई दावा नहीं करेंगे न ही जैविक संसाधनों/व्युत्पन्नियों या अन्य संघटकों पर आवेदक संस्था और राष्ट्रीय जैव विविधता प्राधिकरण के पूर्व अनुमोदन के बिना बौद्धिक संपदा अधिकार का दावा करेंगे ।

हस्ताक्षर-----------------

पदनाम--------------------

कार्यालय की मुहर-------------

उपाबंध 1

उचित और साम्यापूर्ण फायदा बंटाने के विकल्प

निम्नलिखित विकल्पों को या तो एक या अधिक को आवेदक और एनबीए के बीच पारस्परिक रूप से सहमत निबंधनों के आधार पर मामला दर मामला के आधार पर जैव विविधता नियम, 2004 के नियम 20 के उप नियम (3) के उपबंधों के अनुसार लागू किया जा सकेगा । ये विकल्प उपदर्शन की प्रकृति के हैं और अन्य विकल्प जैसा कि एनबीए द्वारा केन्द्रीय सरकार के साथ परामर्श से अनुमोदित किए जाएं , को भी अंगीकार किया जा सकता है:

क. धनीय फायदे विकल्प:

(i) अग्रिम संदाय ;

(ii) एक मुश्त संदाय;

(iii) मुख्य उपलब्धियों पर संदाय;

(iv) स्वामिस्व और उद्‌भूत फायदों को बांटना;

(v) अनुज्ञप्ति फीसों को बांटना;

(vi) राष्ट्रीय, राज्य या स्थानीय जैव विविधता निधियों में अंशदान;

(vii) भारत में अनुसंधान और विकास का वित्त पोषण;

(viii) भारतीय संस्थाओं और कंपनियों के साथ संयुक्त उद्यम;

(ix) सुसंगत बौद्धिक संपदा अधिकारों का संयुक्त स्वामित्व;

ख. गैर धनीय फायदों का विकल्प

(i) सांस्थानिक सक्षमता वर्धन का उपबंध करना जिसके अंतगत धारणीय उपयोग पर प्रशिक्षण अवसंरचना का सृजन और जैविक संसाधनों के संरक्षण और धारणीयता से संबंधित विकास कार्य हाथ में लेना;

(ii) प्रौद्योगिकी का अंतरण या भारतीय संस्थाओं/व्यष्टिकों/निकायों के साथ अनुसंधान और विकास के परिणामों को बांटना;

(iii) प्रौद्योगिकियों के विकास के लिए क्षमताओं का सुदृढीकरण और भारत को प्रोद्योगिकियों का अंतरण और/या भारतीय संस्थाओं/व्यष्टिकों/निकायों के साथ सहयोगकारी अनुसंधान और विकास कार्यक्रम;

(iv) भारत में जैविक संसाधनों के संरक्षण और भरणीयता उपयोग पर शिक्षा और प्रशिक्षण से संबंधित अंशदान/सहयोग;

(v) उत्पादन, अनुसंधान और विकास एककों की अवस्थिति और उस क्षेत्र में प्रजातियों के संरक्षण और परिरक्षण के लिए उपाय जहां जैविक संसाधनों तक पहुंच की गई है, स्थानीय अर्थव्यवस्था में अंशदान और स्थानीय समुदायों के लिए आय सृजन;

(vi) जैविक संसाधनों के संरक्षण और भरणीय उपयोग जिसके अंतर्गत जैविक सूचियां और वर्गीकरण से सुसंगत वैज्ञानिक जानकारी को बांटना भी है;

(vii) भारत में पूर्वीकता आवश्यकताओं की ओर निदेशित अनुसंधान का संचालन जिसके अंतर्गत खाद्य, स्वास्थ्य और जैविक संसाधनों पर ध्यान केन्द्रित करते हुए जीवन यापन सुरक्षा भी है;

(viii) अध्येता वृत्तियों, सहायता और वित्तीय सहायता का भारतीय संस्थाओं/व्यष्टियों को उन क्षेत्रों/जनजातियों/पंथों जो जैविक संसाधनों और पश्चातवर्ती लाभ, यदि कोई है के परिदान में योगदान कर रहे हैं, का उपबंध करना;

(ix) फायदा दावाकर्ताओं को सहायता प्रदान करने के लिए जोखिम पूंजी निधि की स्थापना;

(x) फायदे का दावा करने वालों को धनीय प्रतिकर और अन्य गैर धनीय फायदों का संदाय करना, जैसा कि एनबीए उपयुक्त समझे ।

MINISTRY OF ENVIRONMENT, FORESTS AND CLIMATE CHANGE

(National Biodiversity Authority)

NOTIFICATION

New Delhi, the 21st November, 2014

G.S.R 827.—In exercise of the powers conferred by section 64 read with sub-section (1) of section 18 and sub-section (4) of section 21 of the Biological Diversity Act, 2002 (18 of 2003), hereinafter referred to as the Act, and in pursuance of the Nagoya Protocol on access to genetic resources and the fair and equitable sharing of benefits arising from their utilization to the Convention on Biological Diversity dated the 29th October, 2010, the National Biodiversity Authority hereby makes the following regulations, namely.—

Short title and commencement.—

(1) These regulations may be called Guidelines on Access to Biological Resources and Associated Knowledge and Benefits Sharing Regulations, 2014.

(2) They shall come into force on the date of their publication in the Official Gazette.

1. Procedure for access to biological resources and/ or associated traditional knowledge for research or bio-survey and bio-utilization for research. —

(1) Any person referred to under sub-section (2) of section 3 of the Act, who intends to have access to biological resources and/ or associated traditional knowledge for research or bio-survey and bio-utilization for research shall apply to the National Biodiversity Authority (NBA) in Form I of the Biological Diversity Rules, 2004 for obtaining access to such biological resource and/ or associated knowledge, occurring in India.

(2) The NBA shall, on being satisfied with the application under sub-regulation (1), enter into a benefit sharing agreement with the applicant which shall be deemed as grant of approval for access to biological resource for research referred to in that sub-regulation:

Provided that in case of biological resources having high economic value, the agreement may contain a clause to the effect that the benefit sharing shall include an upfront payment by applicant, of such amount, as agreed between the NBA and the applicant.

2. Procedure for access to biological resources, for commercial utilization or for bio-survey and bio-utilization for commercial utilization. —

(1) Any person who intends to have access to biological resources including access to biological resources harvested by Joint Forest Management Committee (JFMC)/ Forest dweller/ Tribal cultivator/ Gram Sabha, shall apply to the NBA in Form-I of the Biological Diversity Rules, 2004 or to the State Biodiversity Board (SBB), in such form as may be prescribed by the SBB, as the case may be, along with Form 'A' annexed to these regulations.

(2) The NBA or the SBB, as the case may be, shall, on being satisfied with the application under sub-regulation (1), enter into a benefit sharing agreement with the applicant which shall be deemed as grant of approval for access to biological resources, for commercial utilization or for bio-survey and bio-utilization for commercial utilization referred to in that sub-regulation.

3. Mode of benefit sharing for access to biological resources, for commercial utilization or for bio-survey and bio-utilization for commercial utilization.—

(1) Where the applicant/ trader/ manufacturer has not entered into any prior benefit sharing negotiation with persons such as the Joint Forest Management Committee (JFMC)/ Forest dweller/ Tribal cultivator/ Gram Sabha, and purchases any biological resources directly from these persons, the benefit sharing obligations on the trader shall be in the range of 1.0 to 3.0% of the purchase price of the biological resources and the benefit sharing obligations on the manufacturer shall be in the range of 3.0 to 5.0% of the purchase price of the biological resources:

Provided that where the trader sells the biological resource purchased by him to another trader or manufacturer, the benefit sharing obligation on the buyer, if he is a trader, shall range between 1.0 to 3.0% of the purchase price and between 3.0 to 5.0%, if he is a manufacturer:

Provided further that where a buyer submits proof of benefit sharing by the immediate seller in the supply chain, the benefit sharing obligation on the buyer shall be applicable only on that portion of the purchase price for which the benefit has not been shared in the supply chain.

(2) Where the applicant/ trader/ manufacturer has entered into any prior benefit sharing negotiation with persons such as the Joint Forest Management Committee (JFMC)/ Forest dweller/ Tribal cultivator/ Gram Sabha, and purchases any biological resources directly from these persons, the benefit sharing obligations on the applicant shall be not less than 3.0% of the purchase price of the biological resources in case the buyer is a trader and not less than 5.0% in case the buyer is a manufacturer.

(3) In cases of biological resources having high economic value such as sandalwood, red sanders, etc. and their derivatives, the benefit sharing may include an upfront payment of not less than 5.0%, on the proceeds of the auction or sale amount, as decided by the NBA or SBB, as the case may be, and the successful bidder or the purchaser shall pay the amount to the designated fund, before accessing the biological resource.

4. Option of benefit sharing on sale price of the biological resources accessed for commercial utilization under regulation 2.—

When the biological resources are accessed for commercial utilization or the bio-survey and bio-utilization leads to commercial utilization, the applicant shall have the option to pay the benefit sharing ranging from 0.1 to 0.5 % at the following graded percentages of the annual gross ex-factory sale of the product which shall be worked out based on the annual gross ex-factory sale minus government taxes as given below:-

Annual Gross ex-factory sale of product	Benefit sharing component
Up to Rupees 1,00,00,000	0.1 %
Rupees 1,00,00,001<br>up to 3,00,00,000	0.2 %
Above Rupees 3,00,00,000	0.5 %

5. Collection of fees.—

Collection of fees, if levied by Biodiversity Management Committee (BMC) for accessing or collecting any biological resource for commercial purposes from areas falling within its territorial jurisdiction under sub-section (3) of section 41 of the Act, shall be in addition to the benefit sharing payable to the NBA/SBB under these regulations.

6. Procedure for transfer of results of research relating to biological resources.—

(1) Any person who intends to transfer results of research relating to biological resources occurring in or obtained from India, to persons referred to under sub-section (2) of section 3 of the Act for monetary consideration or otherwise, shall—

(a) apply to the NBA in Form II of the Biological Diversity Rules, 2004 for transfer of the results of research relating to biological resources occurring in or obtained from India for any purpose;

(b) provide evidence of approval of NBA for access to the biological resource and/or associated knowledge involved in the research:

Provided that the requirement of evidence under this clause shall not apply to an applicant who is a citizen of India or a body corporate, association or organization which is registered in India and not having any non-Indian participation in its share capital or management;

(c) provide complete information on potential commercial value within the knowledge of the applicant, of the results of research.

(2) The NBA shall, on being satisfied with the application under sub-regulation (1), enter into a benefit sharing agreement with the applicant which shall be deemed as grant of approval for transfer of the results of research referred to in that sub-regulation.

7. Mode of benefit sharing for transfer of results of research. — Applicant shall, in case of transfer of results of research under regulation 6, pay to the NBA such monetary and / or non-monetary benefit, as agreed between the applicant and the NBA:

Provided that in case of monetary benefit received by him, if any, on such transfer, the applicant shall pay to the NBA 3.0 to 5.0% of the monetary consideration.

8. Procedure for obtaining Intellectual Property Rights (IPR). —

(1) Any person who intends to obtain any intellectual property right by whatever name called, in or outside India, for any invention based on any research or information on any biological resources obtained from India, shall make an application to the NBA in Form III of the Biological Diversity Rules, 2004:

Provided that if the applicant is a person referred to under sub-section (2) of section 3 of the Act, he shall provide evidence of approval of NBA for access to the biological resources and/or associated knowledge used in the research leading to the invention:

Provided further that any person applying for any right under the Protection of Plant Varieties and Farmers' Rights Act, 2001 (53 of 2001) shall be exempted from this sub-regulation.

(1) The NBA shall, on being satisfied with the application under sub-regulation (1), enter into a benefit sharing agreement with the applicant which shall be deemed as grant of approval for obtaining IPR.

9. Mode of benefit sharing in IPR. —

(1) The applicant shall, in case of commercializing the IPR obtained, pay to the NBA such monetary and/or non-monetary benefit, as agreed between the applicant and the NBA.

(2) Where the applicant himself commercialises the process/ product/ innovation, the monetary sharing shall be in the range of 0.2 to 1.0% based on sectoral approach, which shall be worked out on the annual gross ex-factory sale minus government taxes.

(3) Where the applicant assigns / licenses the process / product / innovation to a third party for commercialisation, the applicant shall pay to NBA 3.0 to 5.0% of the fee received (in any form including the license / assignee fee) and 2.0 to 5.0% of the royalty amount received annually from the assignee / licensee, based on sectoral approach.

10. Obligations of applicant in the event of commercialisation of IPR. —

(1) An applicant, granted IPR, who is a citizen of India or a body corporate, association or organisation which is registered in India and not having any non-Indian participation in its share capital or management, shall give prior intimation to the concerned SBB for access to biological resources, in the form prescribed by the SBB, and shall comply with such terms and conditions, if any, imposed by the SBB in the interest of promoting conservation and sustainable use.

(2) An applicant, granted IPR, who is a person or a body corporate or an association or an organization referred to under sub-section (2) of section 3 of the Act shall apply in Form I of the Biological Diversity Rules, 2004 to the NBA for access to biological resources.

11. Procedure for transfer of accessed biological resource and/ or associated knowledge to third party for research/ commercial utilization. —

(1) Any person who intends to transfer the biological resources and/or associated knowledge which has been granted access under regulation 1 to a third party for research or for commercial utilization, shall apply to NBA in Form IV of the Biological Diversity Rules, 2004 for transfer to such third party.

(2) The NBA shall, on being satisfied with the application under sub-regulation (1), enter into a benefit sharing agreement with the applicant, which shall be deemed as grant of approval for transfer of accessed biological resource and/ or associated knowledge referred to in that sub-regulation.

12. Mode of benefit sharing for transfer of accessed biological resource and/ or associated knowledge to third party for research/ commercial utilization.—

(1) The applicant shall pay to the NBA such monetary and/ or non-monetary benefit, as agreed between the applicant and the NBA.

(2) Applicant (transferor) shall pay to the NBA 2.0% to 5.0 % (following a sectoral approach) of any amount and/ or royalty received from the transferee, as benefit sharing, throughout the term of the agreement.

(3) In case the biological resource has high economic value, the applicant shall also pay to the NBA an upfront payment, as mutually agreed between the applicant and the NBA.

13. Conducting of non-commercial research or research for emergency purposes outside India by Indian researchers/ Government institutions.—

(1) Any Indian researcher/ Government institution who intends to carry/ send the biological resources outside India to undertake basic research other than collaborative research referred to in section 5 of the Act shall apply to the NBA in Form 'B' annexed to these regulations.

(2) Any Government Institution which intend to send biological resources to carry out certain urgent studies to avert emergencies like epidemics, etc., shall apply in Form 'B' annexed to these regulations.

(3) The NBA shall, on being satisfied with the application under sub-regulation (1) or sub-regulation (2), accord its approval within a period of 45 days from the date of receipt of the application.

(4) On receipt of approval of the NBA under sub-regulation (3), the applicant shall deposit voucher specimens in the designated national repositories before carrying / sending the biological resources outside India and a copy of proof of such deposits shall be endorsed to NBA.

14. Determination of benefit sharing.—

(1) Benefit sharing may be done in monetary and/ or non-monetary modes, as agreed upon by the applicant and the NBA/ SBB concerned in consultation with the BMC/ Benefit claimer, etc. Options for such benefit sharing are provided in Annexure–1.

(2) Determination of benefit sharing shall be based on considerations such as commercial utilization of the biological resource, stages of research and development, potential market for the outcome of research, amount of investment already made for research and development, nature of technology applied, time-lines and milestones from initiation of research to development of the product and risks involved in commercialization of the product:

Provided that special consideration may be given to cases where technologies/products are developed for controlling epidemics/diseases and for mitigating environmental pollution affecting human/ animal/plant health.

(3) The amount of benefit sharing shall remain the same whether the end product contains one or more biological resources.

(4) Where the biological resources of a product are sourced from the jurisdiction of two or more SBBs, the total amount of the accrued benefits shall be shared among them in proportion as decided by the NBA / SBBs concerned, as the case may be.

15. Sharing of benefits.—

(1) Where approval has been granted by the NBA for research or for commercial utilization or for transfer of results of research or for Intellectual Property Rights or for third party transfer, the mode of benefit sharing shall be as under:—

(a) 5.0% of the accrued benefits shall go to the NBA, out of which half of the amount shall be retained by the NBA and the other half may be passed on to the concerned SBB for administrative charges.

(b) 95% of the accrued benefits shall go to concerned BMC(s) and/ or benefit claimers:

Provided that where the biological resource or knowledge is sourced from an individual or group of individuals or organizations, the amount received under this clause shall directly go to such individual or group of individuals or organizations, in accordance with the terms of any agreement and in such manner as may be deemed fit:

Provided further that where benefit claimers are not identified, such funds shall be used to support conservation and sustainable use of biological resources and to promote livelihoods of the local people from where the biological resources are accessed.

(2) Where approval has been granted by State Biodiversity Board under these regulations.—

The sharing of accrued benefits shall be as under.— the SBB may retain a share, not exceeding 5% of the benefits accrued towards their administrative charges and the remaining share shall be passed on to the BMC concerned or to benefit claimers, where identified:

Provided that where any individual or group of individuals or organizations cannot be identified, such funds shall be used to support conservation and sustainable use of biological resources and to promote livelihoods of the local people from where the biological resources are accessed.

16. Processing of applications received by NBA. —

(1) Every application shall be complete in all respects, including all the enclosures referred thereto.

(2) Incomplete applications devoid of any relevant information specifically sought, including ambiguous replies, incomplete disclosure, absence of proof, etc., shall be returned to the applicants.

(3) The time limit specified for processing the applications shall commence only when the application complete in all respects including fee prescribed is received.

(4) Any information specified in the application as confidential shall not be disclosed, either intentionally or unintentionally, to any person not concerned thereto.

(5) While processing the application for access to any biological resource (including plants and/ or animals and/or their parts or genetic material or derivatives), the NBA may consider the following factors, namely:--

Whether the biological resource is –

(i) cultivated or domesticated or wild;

(ii) rare or endemic or endangered or threatened species;

(iii) accessed directly through the primary collectors living in natural habitat or obtained through intermediaries like traders;

(iv) developed or maintained under *ex*-situ conditions;

(v) of high value/ importance to livelihoods of local communities;

(vi) restricted under the Act or any other law for time being in force;

(vii) exempted under section 40 of the Act;

(viii) included in crops listed under Annex I to the International Treaty on Plant Genetic Resources for Food and Agriculture (ITPGRFA), to which India is a contracting party;

(ix) included in the Appendices of the Convention on International Trade on Endangered Species (CITES).

(6) The NBA while taking any decision on the application relating to the use of biological resources and/or knowledge associated thereto may consult through the SBBs, the BMCs within whose jurisdiction the biological resources and/ or the knowledge occur.

(7) The NBA shall reject the application requesting access to biological resources for the reasons specified under rule 16 of the Biological Diversity Rules, 2004.

(8) On receipt of application, the NBA may make enquiries, as it may deem fit, and if necessary, may consult an expert committee constituted for this purpose.

(9) The NBA may upon such enquiry and/or consultation referred to in sub-regulation (8), by order, grant approval or reject the application:

Provided that where the NBA has rejected such application, the reasons for such rejection shall be recorded in writing after giving an opportunity of being heard to the applicant.

(10) Approval granted by the NBA shall be in the form of written agreement duly signed by an authorised officer of the NBA, the applicant and others as applicable:

Provided that the NBA may grant approval without a written agreement for the purposes of conducting of non-commercial research or research for emergency purposes outside India by Indian researchers/ Government institutions under regulation 13.

(11) Based on any complaint or *suo moto,* the NBA may withdraw the approval granted for access and revoke the written agreement on the grounds specified under rule 15 of the Biological Diversity Rules, 2004:

Provided that a copy of the order of such revocation shall be issued to the concerned State Biodiversity Board and the Biodiversity Management Committees for the purposes of prohibition of access.

(12) Where a request has been made by the applicant for withdrawal of his application or the applicant fails to respond to queries of the NBA within the stipulated time, the NBA shall close the applications or initiate action under these regulations as it deems appropriate:

Provided that if the applicant wishes to revive the application, he shall make a fresh application with the requisite fee.

Note: Application forms for access to biological resources and/ or associated knowledge, guidelines for filling them and form of agreements, are available in the NBA website: www.nbaindia.org.

17. Certain activities or persons exempted from approval of NBA or SBB. —

The following activities or persons shall not require approval of the NBA or SBB, namely:--

(a) Indian citizens or entities accessing biological resources and/ or associated knowledge, occurring in or obtained from India, for the purposes of research or bio-survey and bio-utilization for research in India;

(b) collaborative research projects, involving the transfer or exchange of biological resources or related information, if such collaborative research projects have been approved by the concerned Ministry or

Department of the State or Central Government and conform to the policy guidelines issued by the Central Government for such collaborative research projects;

(c) local people and communities of the area, including growers and cultivators of biological resources, and *vaids* and *hakims*, practising indigenous medicine, except for obtaining intellectual property rights;

(d) accessing biological resources for conventional breeding or traditional practices in use in any agriculture, horticulture, poultry, dairy farming, animal husbandry or bee keeping, in India;

(e) publication of research papers or dissemination of knowledge, in any seminar or workshop, if such publication is in conformity with the guidelines issued by the Central Government from time to time;

(f) accessing value added products, which are products containing portions or extracts of plants and animals in unrecognizable and physically inseparable form; and

(g) biological resources, normally traded as commodities notified by the Central Government under section 40 of the Act.

[F. No. NBA/Tech/2/11]

HEM PANDE, Chairman

FORM A

(See regulation 2)

Information to be furnished for use of biological resources by the applicant

Self-disclosure

Common Name of the biological resource proposed to be used: ________________

Scientific Name : ________________

Plants or animals or parts thereof traded : ________________

Specific purpose of access: __________________________

Locations / source from where procured*	Quantity in Kgs	Rate per unit	State Biodiversity Board	Prospective Buyers/Users (if known)

*List of local body/BMCs, if already identified shall be attached.

Undertaking

1. I have read and understood the terms and conditions of ABS guidelines and I undertake to abide by relevant legal provisions applicable to biological resource.

2. I undertake to obtain the approval of the NBA/ SBB before making any change in the stated purpose.

3. I undertake to furnish/ share the relevant records with the NBA/ SBB, as and when required.

4. I further declare that the Information provided in the form is true and correct and I shall be liable for any incorrect/ wrong information and wilful suppression of the facts.

Signature
Name of the trader/ company/manufacturer/ Authorized Representative
Complete Address of the trader/ company/ manufacturer along with phone number and email address

Place
Date

Form B

(See regulation 13)

Conducting of non-commercial research or research for emergency purposes outside India by Indian researchers/Government institutions using the biological resources

1	Name of the Applicant (Indian researcher/ Government Institution)	
2	Complete Address *	
	a. Permanent	
	b. Present	
3	Name and address of Institution in India	
4	Name of the Supervisor or Head of Institution at the place of work in India	
5	Name and contact details of the Institution or organization who shall guide the proposed research / receiving the biological resources.	
6	Details of the Supervisor or Head of Institution or organization who guides the proposed research or recipient of the biological resources	
7	Name of the funding agency supporting the proposed research	
8	Brief description of the research	
9	Details of biological resources proposed to be carried along or sent for the research	
	i. Name of the biological resource (scientific/ common name)	
	ii. Location of collection (Village/Taluk/Dist./State)	
	iii. Quantity required	
	iv. Duration of the research	
10	If it is for emergency purpose, specify details	

* *Attach self-attested address / ID proof such as Aadhaar card/ PAN card / Passport, etc*.

Undertaking

I, _________________ Son/Daughter/Wife/Husband of ______________ aged_____residing at ____________ in _________________ holding a permanent. I.D.No____________(PAN Card/ Aadhaar Card/ Passport, etc,) hereby declare that all the information provided above is correct and true. I hereby affirm that the biological resources shall be used only for the purposes as stated in the application. I shall not share/provide/part/leave behind any biological resource at my collaborator's facility/ laboratory without approval of the NBA. I, along with my supervisor and collaborator, individually and severally declare that we shall not put to commercial utilization, nor shall seek any IPR claim on the biological resources and associated traditional knowledge used in this research/ collaboration. In case such a situation arises we shall apply to National Biodiversity Authority to seek prior approval. Results, process (es), products or other outcomes arising out of this activity shall be shared with the NBA during and/ or upon completion of research intended along with the copy of relevant documents and publications.

Signature:…………………………
Date: ……………………………
Place:…………….……………..

Declaration by the Supervisor / Head of Institution

I, ___________________ working as _____________ in ______________ (Name of institution) confirm that the details provided by Mr/Dr/Mrs/Ms.________________ are true and correct.

Date: …………………
Place:…………………
Signature:…….………………………
Designation:……….……………
Official Seal: ……….……………

Declaration by the Recipient / Collaborator

I, ______________ working as ____________ in _________________(*Name of Institution / Organization*) hereby affirm that I or my institution / organization shall use the biological resources for the purposes as stated in the application and which were sent by………………….. (*Name of the institution*) or being brought by Mr./Dr./Mrs./Ms……………………. The said biological resources shall be destroyed in full after the completion of the studies/ partnership or upon completion of the studies the biological resources shall be sent back to the institution from where the biological resources were received as the case may be. I or the institution I am associated with shall not claim any ownership under instant application nor shall claim any IP Rights over the biological resources, derivatives or other such components without prior approval of the applicant, institution affiliated and the National Biodiversity Authority.

Signature:……..……………………
Designation:………..………………
Official Seal: ………………………

Annexure 1

Fair and equitable benefit sharing options

The following options, either one or more, may be applied in accordance with mutually agreed terms between the applicant and the NBA, on a case by case basis, in accordance with the provisions of sub-rule (3) of rule 20 of the Biological Diversity Rules, 2004. These options are indicative in nature and other options, as approved by the NBA in consultation with the Central Government, may also be adopted:

(a) Monetary benefits options:

(i). Up-front payment;

(ii). One-time payment;

(iii). Milestone payments;

(iv). Share of the royalties and benefits accrued;

(v). Share of the license fees;

(vi). Contribution to National, State or Local Biodiversity Funds;

(vii). Funding for research and development in India;

(viii). Joint ventures with Indian institutions and companies;

(ix). Joint ownership of relevant intellectual property rights.

(b) Non-monetary benefits options:

(i). Providing institutional capacity building, including training on sustainable use practices, creating infrastructure and undertaking development of work related to conservation and sustainable use of biological resources;

(ii). Transfer of technology or sharing of research and development results with Indian institutions/ individuals/entities;

(iii). Strengthening of capacities for developing technologies and transfer of technology to India and/or collaborative research and development programmes with Indian institutions/ individuals/ entities;

(iv). Contribution/ collaboration related to education and training in India on conservation and sustainable use of biological resources;

(v). Location of production, research, and development units and measures for conservation and protection of species in the area from where biological resource has been accessed, contributions to the local economy and income generation for the local communities;

(vi). Sharing of scientific information relevant to conservation and sustainable use of biological diversity including biological inventories and taxonomic studies;

(vii). Conducting research directed towards priority needs in India including food, health and livelihood security focusing on biological resources;

(viii). Providing scholarships, bursaries and financial aid to Indian institutions/ individuals preferably to regions, tribes/ sects contributing to the delivery of biological resources and subsequent profitability if any;

(ix). Setting up of venture capital fund for aiding the cause of benefit claimers;

(x). Payment of monetary compensation and other non-monetary benefits to the benefit claimers as the NBA may deem fit.

Printed by the Manager, Government of India Press, Ring Road, Mayapuri, New Delhi-110064
and Published by the Controller of Publications, Delhi-110054
